# Structural basis for TNA synthesis by an engineered TNA polymerase

**DOI:** 10.1038/s41467-017-02014-0

**Published:** 2017-11-27

**Authors:** Nicholas Chim, Changhua Shi, Sujay P. Sau, Ali Nikoomanzar, John C. Chaput

**Affiliations:** Departments of Pharmaceutical Sciences, Chemistry, and Molecular Biology and Biochemistry University of California, Irvine, CA 92697-3958 USA

## Abstract

Darwinian evolution experiments carried out on xeno-nucleic acid (XNA) polymers require engineered polymerases that can faithfully and efficiently copy genetic information back and forth between DNA and XNA. However, current XNA polymerases function with inferior activity relative to their natural counterparts. Here, we report five X-ray crystal structures that illustrate the pathway by which α-(l)-threofuranosyl nucleic acid (TNA) triphosphates are selected and extended in a template-dependent manner using a laboratory-evolved polymerase known as Kod-RI. Structural comparison of the apo, binary, open and closed ternary, and translocated product detail an ensemble of interactions and conformational changes required to promote TNA synthesis. Close inspection of the active site in the closed ternary structure reveals a sub-optimal binding geometry that explains the slow rate of catalysis. This key piece of information, which is missing for all naturally occurring archaeal DNA polymerases, provides a framework for engineering new TNA polymerase variants.

## Abstract (web summary)

The laboratory-evolved polymerase Kod-RI catalyzes α-L-threose nucleic acid (TNA) synthesis. Here, the authors present Kod-RI crystal structures that give insights into how TNA triphosphates are selected and extended in a template-dependent manner, which will help to engineer improved TNA polymerases for synthetic genetics applications.

## Introduction

Synthetic genetics is an emerging field of science that aims to extend the principles of heredity and evolution to nucleic acid polymers with backbone structures that are distinct from those found in nature^1^. Collectively referred to as xeno-nucleic acids or XNA^2^, these polymers have unique physicochemical properties that often include strong resistance to degradative enzymes and duplex structures that adopt a range of helical geometries^3^. By engineering polymerases to synthesize and recover genetic information encoded in XNA, researchers are developing complex molecular systems that are capable of undergoing Darwinian evolution in response to imposed selection constraints^4^. These studies, which expand our ability to store, propagate, and evolve genetic information, have profound implications for biotechnology, molecular medicine, and the origin of life^5^.

**Fig. 1:**
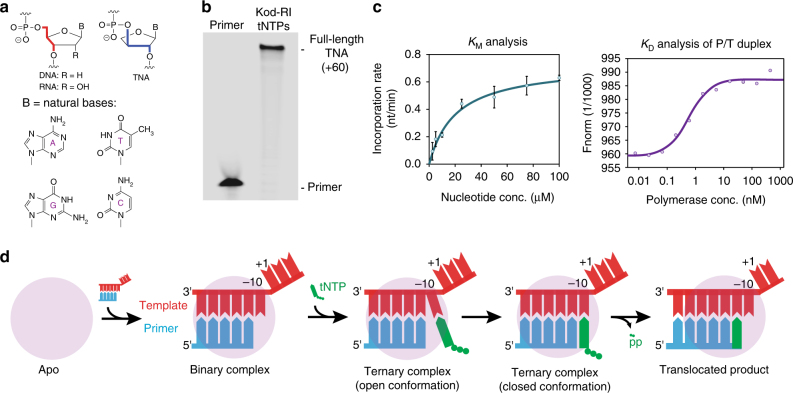
TNA synthesis by Kod-RI. **a** Molecular structures comparing TNA to DNA and RNA. **b** Denaturing PAGE showing TNA synthesis on a library of degenerate DNA templates. **c** Functional analysis of TNA substrate and primer template binding. Error bars represent the average of three independent trials. **d** Schematic view of the TNA synthesis pathway where each cartoon image represents an elucidated structure described in this study

To date, five different XNA polymers with non-ribose sugars (1,5-anhydrohexitol nucleic acid (HNA), arabino nucleic acid (ANA), 2′-fluoro-arabino nucleic acid (FANA), cyclohexenyl nucleic acid (CeNA) and α-l-threose nucleic acid (TNA)) have achieved successful replication in a Darwinian evolution system^6–9^. Of these, TNA is considered to be the most structurally diverse, because it has a backbone repeat unit that is one atom shorter than that of DNA or RNA (Fig. 1a)^10^. By comparison, all other XNAs that are capable of Darwinian evolution maintain the same six-atom backbone repeat unit found in natural DNA and RNA^1^. Remarkably, despite this difference, TNA is capable of forming stable antiparallel Watson–Crick duplex structures with itself and with complementary strands of DNA and RNA^10, 11^. Solution NMR studies reveal that duplex formation in either the self-pairing mode (TNA/TNA) or cross-pairing mode (TNA/DNA or TNA/RNA) occurs through an A-like helical geometry that is templated by a rigid TNA backbone^12, 13^. More recently, stability assays performed under harsh biological conditions demonstrate that TNA is refractory to nuclease digestion^14^. This feature, coupled with the ability to undergo Darwinian evolution in vitro^8^, make TNA a promising candidate for diagnostic and therapeutic applications that require high biological stability^15^.

TNA synthesis is made possible by Kod-RI, a laboratory-evolved polymerase that derives from a replicative B-family polymerase isolated from the archaeal hyperthermophilic species *Thermococcus kodakarensis* (Kod)^16^. In addition to the 3′,5′-exonuclease silencing mutations D141A and E143A, Kod-RI carries the TNA synthesis mutations A485R and E664I (Supplementary Fig. 1) that were identified using the microfluidic strategy of droplet-based optical polymerase sorting, which allows for rapid screening of engineered polymerases in uniform microcompartments^17^. Kod-RI is the most efficient TNA polymerase developed to date^16^, and exhibits 5-fold faster primer-extension efficiency (3 h vs. 15 h, respectively) and ~20-fold higher fidelity (four errors per 1000 nucleotide incorporations vs. 70 errors, respectively) than Therminator DNA polymerase (9°N, A485L), which was previously used for TNA synthesis^18^.

Although more than 30 years have passed since the first polymerase structure was solved by X-ray crystallography^19^, no structural information is available for an archaeal polymerase with a primer–template and nucleoside triphosphate bound in the enzyme-active site. Since archaeal polymerases are common enzymes for many biotechnology applications^20^, open and closed ternary structures would help evaluate the mechanism of natural DNA synthesis relative to other polymerase families. However, the crystal structures of XNA polymerases themselves are even more valuable, as these structures would provide insights into the limitations of existing XNA polymerases. In their absence, structural information about the ternary complex must be derived from distantly related viral (RB69 Pol and Phi29 Pol) and eukaryotic polymerases (Pols α, δ, and ε), which share only ~20% identity with archaeal B-family polymerases^21–25^.

Here, we describe a structural approach that was taken to evaluate the pathway by which a laboratory-evolved polymerase is able to synthesize unnatural TNA polymers on natural DNA templates. The collection of five X-ray crystal structures details an ensemble of intermolecular interactions and conformational changes that allow Kod-RI to promote TNA synthesis. Close inspection of the enzyme-active site in the closed ternary structure trapped in a pre-catalytic state reveals a sub-optimal binding geometry for the incoming TNA triphosphate. This key piece of information explains the slow rate of Kod-RI mediated TNA synthesis relative to DNA synthesis by natural Kod DNA polymerase. Together, the set of X-ray crystal structures offer insight into the structural plasticity of DNA polymerases and provide a framework that can be used to guide the engineering of new TNA polymerase variants that function with improved catalytic activity.

## Results

### Function

Kod-RI is a DNA-dependent TNA polymerase that is able to transcribe individual strands or large libraries of degenerate DNA sequences into TNA (Fig. 1b)^16^. This property, which is remarkable considering the backbone structure of TNA relative to DNA and RNA, has enabled the evolution of TNA aptamers from unbiased pools of random sequences^26^. The enzyme functions by a primer-extension mechanism in which a primer strand (DNA or TNA) annealed to a DNA template is extended with chemically synthesized TNA triphosphates. Previous analyses indicate that Kod-RI functions with a modest rate of ~1 nucleotide per minute^16^, which is ~10,000-fold slower than the rate of DNA synthesis by wild-type Kod DNA polymerase^27^. Affinity measurements made on the primer–template (P/T) complex and TNA triphosphates reveal that Kod-RI has a *K*_D_ of ~0.6 nM for the P/T duplex and a *K*_M_ of ~15 μM for tNTP substrates (Fig. 1c). These values, which are within the range of natural archaeal B-family DNA polymerases^28^, led us to speculate that the slow rate of TNA synthesis is due to an imperfect active site that positions tNTP substrates in a geometry that is sub-optimal for phosphodiester bond formation.

### Crystallization

**Fig. 2:**
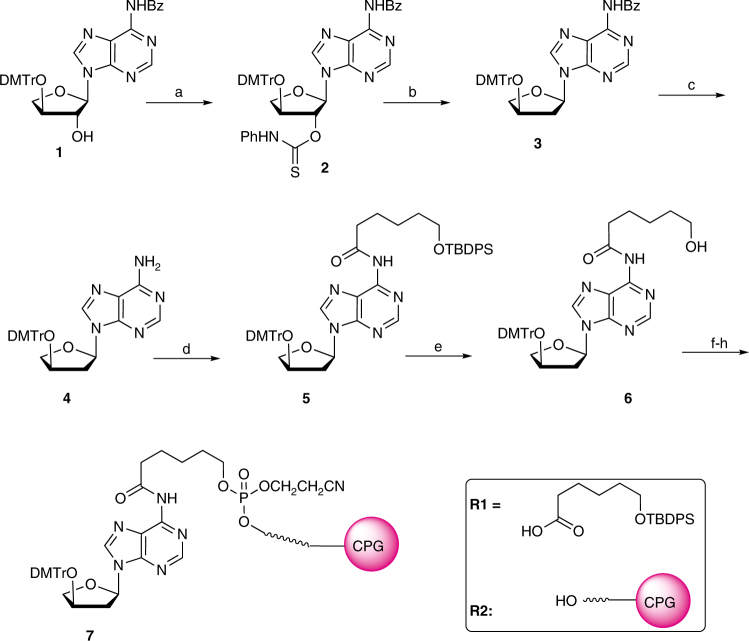
Chemical synthesis scheme of 2′-deoxy-α-l-threofuranosyl adenosine-modified CPG (Compound 7). Reagents: **a** PhNCS, NaH, THF, rt, 2.5 h, 80%; **b** AIBN, Bu_3_SnH, toluene, 100 °C, 45 m, 86%; **c** K_2_CO_3_, MeOH, 72%; **d R1**, PyBOP, DIPEA, DCE, 43%; **e** TBAF/THF, 70%; **f** 2-cyanoethyl-*N,N*-diisopropylchlorophosphoramidite, DIPEA, DCM; **g R2**, EtS-tetrazole, MeCN; **h** I_2_/Py-water

To provide structural insights into the mechanism of TNA synthesis, protein crystals of Kod-RI were grown under conditions that were designed to capture four main steps in the TNA synthesis pathway (Fig. 1d), namely P/T binding, nucleoside triphosphate binding, catalysis, and translocation. Critical to this effort was the chemical synthesis of a chain-terminating primer bearing a 2′-deoxy-α-l-threofuranosyl adenosine residue (tA^d^) at the 3′ end. Controlled pore glass (CPG) functionalized with a tA^d^ analog was obtained in 8 synthetic steps (Fig. 2) from a known dimethoxytrityl-protected α-l-threofuranosyl adenosine nucleoside^29^. Solid-phase synthesis was then used to construct the DNA primer (P2, Supplementary Table 1) from the tA^d^-modified CPG resin. A second primer (P1, Supplementary Table 1) bearing an unmodified TNA adenosine residue (tA) was similarly constructed using tA-modified CPG. The α-l-threofuranosyl adenosine 3′-triphosphate (tATP) required for crystallization was obtained in 12 steps from l-ascorbic acid using known methodology^29, 30^.

Although Kod-RI crystallized in the presence of tATP and magnesium, no electron density was observed for either the bound triphosphate or magnesium ions. Similar results have been reported by others for crystallization trials conducted on the wild-type Kod polymerase and related homologs^31, 32^. In an effort to overcome this problem, we explored a variety of crystallization conditions, which included variations in tATP concentration, soaking times, and metal ion additives. After extensive optimization, we successfully obtained open and closed ternary structures with clear electron density for the TNA substrate and bound magnesium ions in the enzyme-active site. The condition that proved most successful involved soaking co-crystals grown in the presence of tATP with excess tATP and/or magnesium ions just prior to freezing. Specifically, the open ternary structure required a 20 min soak with 0.2 mM tATP, whereas the closed ternary structure required a 45 min soak with 2 mM tATP and 20 mM MgCl_2_. Although the precise reason for why the ternary structures required additional soaking is unclear, we speculate that it could be due to an active site pocket that allows for the rapid exchange of nucleotide substrates.

### Structures

Five structures spanning a resolution limit of 2.05–3.2 Å were solved by molecular replacement (Table 1 and Supplementary Fig. 2). We used the apo and binary forms of wild-type Kod DNA polymerase (PDB ID: 1WNS and 4K8Z), respectively^31, 33^, as the search models for apo and binary Kod-RI structures. Structures obtained for the open ternary and translocated product used an early binary Kod-RI structure as the search model, while the closed ternary structure used an early open ternary structure as the search model. The final apo Kod-RI structure contained unbuilt regions in the thumb subdomain due to poor electron density. The remaining four structures (i.e., binary, open and closed ternary, and translocated product) feature Kod-RI bound to a P/T duplex, where the primer contained one or more tA residues at the 3′ end. Due to poor electron density in the triphosphate tail, tA was modeled as the substrate for the open ternary structure. For the closed ternary structure, tATP was modeled as the substrate.

**Table 1:** Data collection and refinement statistics

	Apo	Binary	Ternary (open)	Ternary (closed)	Translocated product
*Data collection*					
Space group	*P*2_1_2_1_2_1_	*P*22_1_2_1_	*P*22_1_2_1_	*P*22_1_2_1_	*P*22_1_2_1_
Cell dimensions					
* a*, *b*, *c* (Å)	72.8, 110.6, 111.8	66.1, 110.8, 149.1	66.5, 112.6, 145.5	72.3, 107.4, 147.7	69.1, 110.9, 151.1
* α*, *β*, *γ* (°)	90.0, 90.0, 90.0	90.0, 90.0, 90.0	90.0, 90.0, 90.0	90.0, 90.0, 90.0	90.0, 90.0, 90.0
Resolution (Å)	72.81–2.80 (2.95–2.8)	88.93–3.0 (3.18–3.0)	66.55–2.72 (2.85–2.72)	50.0–3.2 (3.31–3.2)	89.41–2.05 (2.1–2.05)
*R*_merge_	0.319 (1.157)	0.0912 (0.779)	0.0738 (0.575)	0.275 (1.100)	0.034 (0.789)
CC1/2	0.659 (0.31)	0.99 (0.483)	0.992 (0.559)	0.981 (0.701)	0.998 (0.594)
*I* / *σI*	7.4 (0.7)	5.8 (1.0)	7.6 (1.5)	9.7 (1.5)	11.0 (1.1)
Completeness (%)	86.4 (83.2)	99.7 (98.8)	97.4 (97.9)	99.6 (99.2)	94.9 (90.6)
Redundancy	10.5 (10.7)	12.0 (11.6)	3.7 (3.7)	7.0 (6.5)	11.3 (9.6)
*Refinement*					
Resolution (Å)	49.57–2.8 (2.90–2.80)	88.93–3.0 (3.12–3.0)	49.1–2.72 (2.79–2.72)	43.42–3.2 (3.29–3.2)	45.85–2.05 (2.11–2.05)
No. reflections	19,774 (1872)	22,538 (2156)	29,340 (2891)	19,530 (1919)	69,934 (6584)
*R*_work_/*R*_free_	0.268/0.317 (0.426/0.446)	0.236/0.283 (0.325/0.366)	0.214/0.268 (0.330/0.369)	0.256/0.295 (0.366/0.386)	0.190/0.235 (0.365/0.446)
No. atoms	5742	6743	6746	6680	7149
Protein	5742	6214	6214	6120	6210
Duplex/tATP	–	529	506/20	528/29	592
Solvent/Mg^2+^	–	–	5/1	1/2	347/0
B-factors	111.8	70.3	53.1	101.3	53.4
Protein	111.8	68.9	52.2	99.58	52.7
Duplex/tATP	–	86.8/–	64.8/45.9	122.9/84.0	60.4
Solvent/ Mg^2+^	–	–	109.5/44.6	64.8/71.6	54.5/0
Rms deviations					
Bond lengths (Å)	0.002	0.007	0.004	0.002	0.013
Bond angles (°)	0.58	0.85	0.61	0.54	1.15

### Architecture

**Fig. 3:**
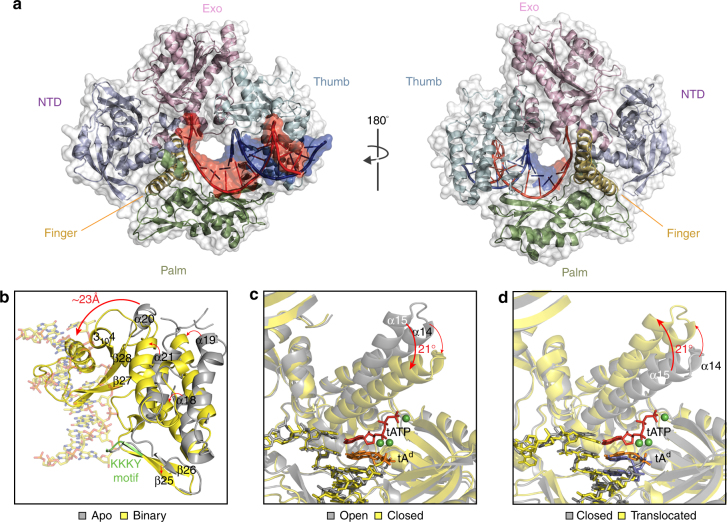
Structure and conformational changes. **a** Global architecture of Kod-RI bound to a P/T duplex. Front (left) and back (right) views of Kod-RI colored by domain with the template and primer strands shown in red and blue, respectively. Structural changes observed during primer–template binding **b**, nucleoside triphosphate binding **c**, and translocation **d**. The 2′-deoxy threose adenosine (tA^d^) residue at the 3′-terminus of the DNA primer is colored in orange to distinguish it from the DNA portion of the P/T duplex (yellow sticks). Magnesium ions appear as green spheres. In the translocated structure **d**, the TNA residues are colored blue

Consistent with all known structures of B-family polymerases, Kod-RI adopts a disk-shaped architecture that encompasses the N-terminal (NTD), exonuclease (Exo), and catalytic domains (Fig. 3a)^34^. The catalytic domain is further divided into the palm, finger, and thumb subdomains. The P/T duplex is bound in a groove defined by the palm and thumb subdomains, making contacts to 9 base pairs in the P/T duplex. Interaction maps created for the binary and ternary structures reveal that the duplex is primarily recognized by contacts made to the phosphodiester backbone, with only a small number of direct contacts being made to the sugar and nucleobase moieties (Supplementary Fig. 3a–d). All of the sugar and nucleobase contacts occur through the minor grove, which is consistent with the propensity for B-family polymerases to accept modified nucleotides bearing functional groups at the C5 pyrimidine and C7 deazapurine positions^35^. In addition, many residues responsible for recognizing the P/T duplex are highly conserved among

B-family polymerases, including the sequence motif KKKY (residues 591–594, Fig. 3b), which is thought to stabilize the B-form helix by bringing the primer and template closer together^36^. The unpaired region of the template is stabilized by residues from the NTD and exo domains, which cause an abrupt kink in the template at the +1 position. These interactions are all consistent with the high resolution binary structures previously solved for Kod and 9°N^31^.

### Conformational changes

Because we were able to solve all five of the polymerase structures that define the TNA synthesis pathway, it was possible to study the conformational changes that facilitate TNA synthesis. Comparative structural analyses identified three major conformational changes between the set of five Kod-RI structures. The first conformational change arises when the apo form of the polymerase binds the P/T duplex to form the binary complex (Fig. 3b). Upon P/T binding, the thumb subdomain transitions from an ensemble of poorly defined conformations to a well-ordered binary structure. One striking example of positional rearrangement is helix α20, which shifts ~23 Å to bind the minor groove face of the P/T duplex (Fig. 3b). In addition, several other secondary structural elements (e.g., β26–28 and 3_10_4) not visible in the apo structure become visible in the binary complex (Fig. 3b). These structural changes signify the importance of the thumb subdomain in P/T binding.

The second major conformational change involves formation of the ternary complex with the P/T duplex and tATP substrate bound in the enzyme-active site. This step involves insertion of the tNTP substrate into the active site pocket followed by a closing of the finger subdomain onto the thumb subdomain to form the catalytically relevant closed ternary structure. Structural alignment of the binary and open ternary complexes (rmsd 0.6 Å) reveals that the binary and open ternary structures are identical (Supplementary Fig. 4), implying that the binary complex can accommodate a tNTP substrate without the need for structural change. This observation is consistent with previous kinetic data showing that dNTPs diffuse directly into the active site of B-family polymerases^37^, rather than occupying a pre-insertion site as has been observed for some A-family polymerases^38^.

**Fig. 4:**
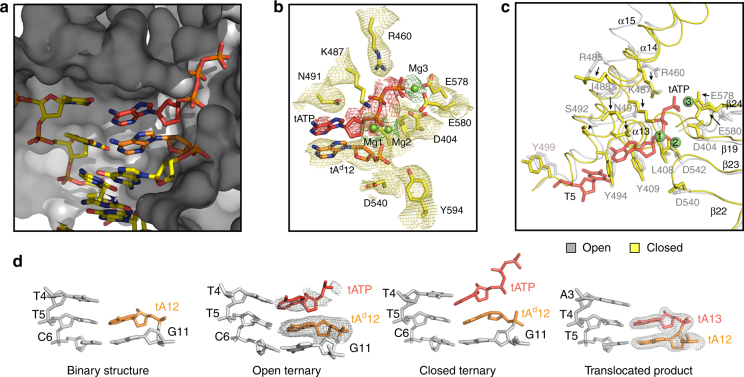
Active site pocket. **a** Surface representation of the enzyme-active site in the closed ternary conformation of Kod-RI. **b** Superimposed on the stick model from (A) is a 2Fo–Fc composite omit map (yellow) contoured at 1.0*σ* for the conserved residues and tA^d^12 and two simulated annealing Fo–Fc omit maps contoured at 2.0*σ* for tATP and the three magnesium ions (red and green, respectively). **c** Structural differences observed between the open and closed conformations of the ternary complex. **d** Stick models comparing the Watson–Crick base pairing geometry for a portion of the P/T duplex. Simulated annealing Fo–Fc omit maps contoured at 3.0*σ* and 4.0*σ* are shown for the open ternary complex and the translocated product, respectively

The initial tATP binding event is followed by a major conformational change (Fig. 3c) in which the finger subdomain closes upon the nucleoside triphosphate. Alignment of the open and closed ternary complexes (rmsd 2.5 Å) reveals that Kod-RI undergoes a substantial conformational change to achieve the catalytically relevant state. For this transition, helix α15 in the finger subdomain tilts 21° inward to form the closed ternary complex in which tATP is trapped in a pre-catalytic state by the 2′-deoxy-tA residue on the primer. In addition, numerous residues in the finger (e.g., Arg^460^, Lys^464^, Lys^487^, and Asn^491^) and palm (e.g., Asp^404^, Asp^540^, Asp^542^, Glu^578^, and Glu^580^) subdomains undergo repositioning to promote substrate recognition and catalysis (Fig. 4c).

Following catalysis, the polymerase experiences a third conformational change whereby the finger subdomain reopens and the polymerase translocates to the next position on the template (Fig. 3d). This last step completes the cycle of nucleotide addition by enabling the polymerase to move from position 0 to position −1 on the DNA template. The structural changes observed between the closed ternary complex and translocated product are similar to the changes observed between the open and closed ternary complexes (rmsd 2.5 Å), which is expected as the finger subdomain will open and close an equal distance between each cycle of nucleotide catalysis.

### Active site analysis

The active site pocket, which encompasses the nascent T4:tATP base pair, is primarily formed by residues Ile^488^, Asn^491^, Ser^492^, Tyr^494^, Gly^495^, Gly^498^, and Tyr^499^ in the finger subdomain and residues Leu^408^ and Tyr^409^ in the palm subdomain (Fig. 4a, b). Three highly conserved carboxylate groups (Asp^404^, Asp^540^, and Asp^542^) mark the polymerase-active site^34^. Interestingly, the steric gate residue Tyr^409^ does not interact with the threose sugar of the incoming tATP substrate (~4.0 Å away) even though this position is known to discriminate against ribonucleoside triphosphates in analogous wild-type polymerases (Supplementary Fig. 5)^39^. Electron density maps indicate that tATP is tightly bound in the closed ternary conformation (Fig. 4b), but only weakly bound in the open conformation (Fig. 4d). This observation is expected based on the location of the finger subdomain in the open and closed ternary structures.

Three magnesium ions are observed in the active site of the closed ternary complex. Two of these (Mg_2_ and Mg_3_) adopt positions that are structurally identical to other B-family polymerases whose ternary structures have been solved by X-ray crystallography (Fig. 4b, c)^21, 24^. A third magnesium ion (Mg_1_), located between the α-phosphate of tATP and the primer strand (Fig. 4b, c), is responsible for aligning the 2′-hydroxyl group on the TNA primer for nucleophilic attack on the tNTP substrate. This metal ion lies 3.2 Å from the C2′ atom of the primer strand and 2.5 Å from the α-phosphate on the incoming tATP substrate. A simulated annealing omit map for tATP reveals that the adenine base is highly flexible relative to the triphosphate tail and threose sugar (Fig. 4b). Nucleobase flexibility is due to an active site pocket that is not fully optimized for the smaller size of the tNTP substrate (Fig. 4a). Unlike DNA, TNA lacks a 5′-methylene carbon, which would facilitate stronger Watson–Crick base pairing by bringing the nucleoside triphosphate closer to the templating base. Presumably, these interactions could be strengthened by mutations in the thumb subdomain that better constrain the tNTP substrate in the active site pocket.

### Base pair geometry

Computational analysis of the Kod-RI structures containing a P/T duplex revealed significant deviations in the planarity of the base pair at the active site^40^. In particular, the buckle and propeller parameters for the T5:tA12 base pair of the binary complex are ~−22° and ~18°, respectively (Fig. 4d, Supplementary Table 2). These distortions are recapitulated in the nascent T4:tATP base pair of the open and closed ternary complexes and the non-planar geometry propagates to the T5:tA12 and C6:G11 base pairs (Supplementary Table 2). However, following a single turnover event, the base pair geometry returns to a normal planar conformation as evidenced in the translocated structure (Fig. 4d), indicating the sub-optimal base pair geometry observed in the pre-catalytic state is corrected following the chemical bond forming step.

Similar base pair analyses performed on the ternary structures from known viral and eukaryotic B-family polymerases bound to dNTPs reveals that the base pair distortions observed for Kod-RI are distinct and severe relative to natural B-family polymerases (Supplementary Table 3)^21–25^. In all cases, base pair planarity is maintained throughout the duplex, including the incoming nucleoside triphosphate, which stacks directly on the 3′ end of the primer strand. One minor deviation from this trend is the binary structure of wild-type Kod DNA polymerase bound to an all-DNA P/T duplex, which exhibits buckle and propeller distortions at T5:A12 (Supplementary Table 2)^31^. Whether this distortion is typical for Kod DNA polymerase is difficult to assess in the absence of more structural information, most notably a ternary structure for the wild-type polymerase.

### TNA synthesis mutations

**Fig. 5:**
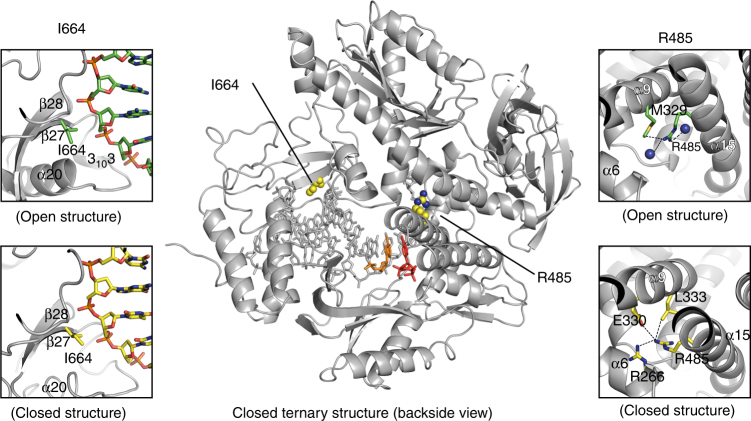
TNA gain-of-function mutations. Closed ternary Kod-RI structure depicting the mutant residues Arg485 and Ile664 as space-filing models (center). Conformational differences observed between the open and closed forms of the ternary structure are depicted for Ile664 (left insets) and Arg485 (right insets). Residues observed in the open and closed conformations are drawn as yellow and green sticks, respectively

Kod-RI differs from natural Kod DNA polymerase by the mutations A485R and E664I, which were identified by directed evolution as amino acid changes that confer TNA synthesis activity on the natural polymerase scaffold^17^. Both mutations are located outside the active site pocket and mutational analysis suggests that the two residues perform independent functions^16^. Arg^485^ is located on helix α15 in the finger subdomain (Fig. 5). In the closed ternary complex, Arg^485^ adopts an upward facing position with respect to the helix that interacts with residues Arg^266^, Glu^330^, and Leu^333^ located on helices α6 and α9 of the Exo domain while this residue in each of the open conformation structures (i.e., binary, open, and translocated), is oriented in the downward position along the helix as shown in Supplementary Fig. 6. Comparison of the binary structures for Kod-RI and natural Kod DNA polymerase, reveals that the bulky Arg^485^ mutation causes helix α15 to bend toward the primer–template duplex (Supplementary Fig. 7). We postulate that this structural perturbation enables the recognition of TNA substrates by altering the shape of the active site pocket.

By contrast, Ile^664^ is located on strand β27 in the thumb subdomain (Fig. 5). This mutation contacts the phosphodiester backbone at nucleotide positions +5 and +6 on the P/T duplex. Holliger and co-workers have identified position 664 as a key site for the processive synthesis of non-cognate nucleic acid polymers^41^. In one example, variants of a DNA polymerase isolated from *Thermococcus gorgonarius* where found to promote efficient RNA synthesis on DNA templates^41^. While the precise functional role of Ile^664^ remains unknown, we speculate that this mutation reduces stringent recognition of the natural P/T duplex by replacing a critical electrostatic interaction with a less discriminating hydrophobic side chain.

## Discussion

Archaea constitute one of the three major evolutionary lineages of life^42^. These organisms exist in a broad range of habitats that include harsh environments, such as hot springs and salt lakes, as well as milder areas consisting of soils, oceans, and marshlands. Because of their ability to withstand high temperatures and organic solvents, enzymes isolated from thermophilic archaea have been exploited in many biotechnology applications^43^. Indeed, numerous examples now exist where archaeal B-family DNA polymerases have been shown to accept chemically modified nucleotides bearing alternative functionality at the sugar or nucleobase moieties^44, 45^ and these enzymes are often used as the starting point for the directed evolution of XNA polymerases^6, 17^. However, despite a prominent role in nature and biotechnology, there exists a paucity of structural information for this important class of DNA polymerases.

Here, we address this shortcoming by providing a series of X-ray crystal structures that describe how a laboratory-evolved polymerase is able to synthesize unnatural TNA polymers on natural DNA templates. This body of work produced X-ray crystal structures of the apo, binary, open ternary, closed ternary, and translocated product of a laboratory-evolved polymerase. Although apo and binary structures have been determined for a limited number of hyperthermophilic B-family polymerases^21–25, 34, 46–48^, the elusive ternary structure remained an outstanding challenge in the field^31, 32^. Critical to our success was the chemical synthesis of a chain-terminating primer bearing a 2′-deoxy-α-l-threofuranosyl adenosine residue that allowed us to trap Kod-RI in the pre-catalytic state. This analog, coupled with an exhaustive search of appropriate crystallization conditions, provided the chemical basis for obtaining open and closed ternary structures with clear electron density for the incoming TNA substrate and magnesium ions.

**Fig. 6:**
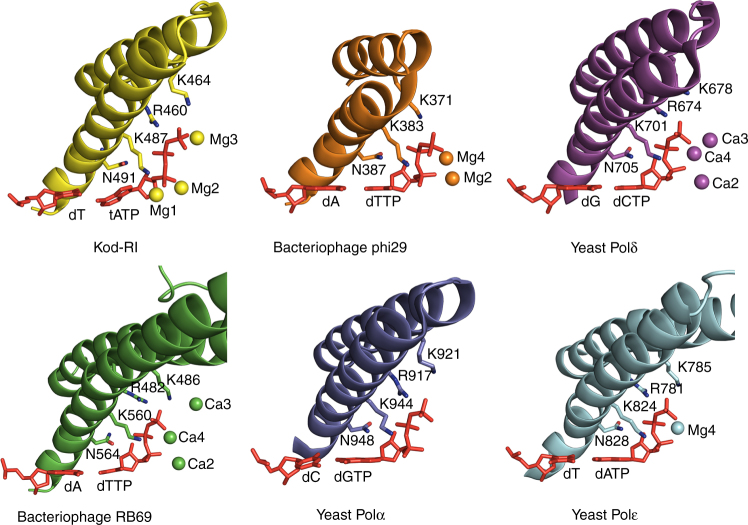
Structural comparison of the finger subdomain for B-family polymerases. Conserved finger subdomain residues across archaeal (Kod-RI), viral (RB69, PDB: 1IG9 and Phi29, PDB: 2PYL), and eukaryotic (Pol α PDB: 4FYD, Pol δ PDB: 3IAY, and Pol ε PDB: 4M8O) polymerases interacting with the nascent base pair. In Kod-RI, Mg1 is novel, whereas Mg2 and Mg3 are observed in other B-family polymerases

The open and closed ternary structure of Kod-RI bound to a primer–template duplex and TNA triphosphate reveals a sub-optimal geometry for the incoming nucleoside triphosphate that is characterized by severe buckle and propeller distortions to the nascent base pair. The sub-optimal binding geometry, which extends to the divalent metal ions, likely accounts for the slow rate of catalysis observed for Kod-RI. By comparison, the ternary structures of distantly related viral and eukaryotic B-family polymerases exhibit a co-planar geometry for the nascent base pair with divalent metal ions that are ideally positioned for the subsequent chemical bond forming step (Fig. 6). However, this result is not surprising when one considers the limited evolutionary history of Kod-RI relative to natural polymerases^17^, which have benefited from billions of years of natural selection. The fact that engineered polymerases are able to synthesize artificial genetic polymers with backbone structures that are distinct from those found in nature is a remarkable achievement that can be further improved by directed evolution.

Moving forward, structural information available in the closed ternary complex provides an important framework for generating new TNA polymerase variants that function with increased catalytic activity. Future efforts aimed at improving the geometry of the nascent base pair would benefit from studies that focus on primary and secondary shell residues in the enzyme-active site. This could, for example, include the mutagenesis of nearby residues in the thumb subdomain as well as possible residues in the finger and palm regions that may be required for compensatory reasons. As these studies continue, efforts to establish a more comprehensive view of natural and engineered archaeal polymerases are warranted and would benefit from high resolution X-ray crystal structures for three important classes of ternary polymerases that have yet to be solved: (i) wild-type polymerase bound to natural dNTP; (ii) wild-type polymerase bound to tNTP; and (iii) Kod-RI bound to natural dNTP. In addition, these efforts would also benefit from binary and ternary structures in which the primer strand of the P/T duplex is composed entirely of TNA. Structural information of this type would make it possible to better evaluate the slow rate of TNA synthesis relative to natural DNA synthesis by the wild-type polymerase.

In summary, structural analysis of the TNA synthesis pathway provides critical insights into the constraints of a laboratory-evolved polymerase. This approach of directed evolution and structure determination provides important clues that can be used to guide the development of future XNA polymerases. In the future, it will be interesting to see how well molecular evolution, either alone or guided by computational design, can close the gap in catalytic activity between engineered polymerases and their natural counterparts. Such efforts open the door to a vast new world of synthetic genetics, where sequence-defined synthetic polymers can be used to create new tools for biotechnology and medicine, and possibly even improve our understanding of the origin of life.

## Methods

### Synthetic procedures

All reagents and solvents of highest purity were purchased from commercial suppliers and used without further purification. Reactions were run under an inert atmosphere of nitrogen or argon, unless otherwise indicated. Thin-layer chromatography (TLC) was performed using Silica Gel 60 F254-coated glass plates (0.250 mm thickness) and visualization was performed with UV irradiation. Chromatography was accomplished with SiliaFlash P60 (230–400 mesh) silica gel. Solutions in organic solvents were dried under reduced pressure using a Buchi rotary evaporator.

NMR spectra were collected on a Bruker DRX-400 (400 MHz^1^H) equipped with QNP probe or a Bruker DRX-500 instrument equipped with a TCI cryogenic probe (500 MHz^1^H, 125.7 MHz^13^C) at 298 K. Spectra are internally referenced to residual solvent signals (CDCl_3_ is referenced to 7.26 ppm for^1^H and 77.16 ppm for^13^C). Chemical shifts are reported in ppm, and coupling constants (*J*) are rounded to integer or half-integer and reported in Hz. High-resolution mass spectrometry was performed by the University of California, Irvine Mass Spectrometry Center.

### Chemical synthesis

*N* ^*6*^-Benzoyl-9-[3′-*O*-[(4′,4″-dimethoxytriphenyl)methyl]-2′-deoxy-α-l-threofuranosyl]adenine (**3**)^49^. To a solution of the protected TNA nucleoside **1** ^29^ (1.5 g, 2.3 mmol) in anhydrous THF (20 mL) was added phenylisothiocyanate (0.4 mL, 3.2 mmol) and 60% NaH in mineral oil (200 mg, 5 mmol) and the reaction was left stirring for 2.5 h at 24 °C. After complete consumption of the starting material, the reaction was quenched by addition of 1 mL water and concentrated under reduced pressure. The residue was suspended in EtOAc (30 mL), sequentially washed with water (15 mL) and brine (15 mL), dried over MgSO_4_, and evaporated to dryness. The pure product (**2**) was obtained as a white foam (1.47 g, 80%) after silica gel chromatography (50–100% EtOAc-Hexane containing 1% TEA).

Next, to a solution of **2** (1.45 g, 1.86 mmol) in anhydrous toluene (20 mL) was added to a solution of 0.2 M AIBN in toluene (10 mL, 20 mmol) and 1 M Bu_3_SnH in cyclohexane (3.5 mL, 3.5 mmol). After heating for 45 min at 100 °C, the mixture was evaporated and purified by silica gel chromatography (50–100% EtOAc-Hexane containing 1% TEA). The pure product (**3**) was obtained as a solid foam (1.0 g, 86%).^1^H NMR (400 MHz, CDCl_3_) δ 8.97 (brs, 1 H, NH), 8.80 (s, 1 H, H2), 8.50 (s, 1 H, H8), 8.03 (m, 2 H, Bz), 7.61 (m, 1 H, Bz), 7.53 (m, 2 H, Bz), 7.40–7.20 (m, 9 H, DMT), 6.81 (d, 4 H, DMT), 6.35 (dd *J* = 2.0, 7.5 Hz, 1 H, H1′,), 4.51 (m, 1 H, H3′), 3.85–3.70 (m, 8 H, 2OCH_3_, H4a′, H4b′), 2.40–2.30 (m, 1 H, H2a′) 2.13 (m, 1 H, H2b′).

9-[3′-*O*-[(4′,4″-dimethoxytriphenyl)methyl]-2′-deoxy-α-l-threofuranosyl]adenine (**4**). To a solution of **3** (400 mg, 0.64 mmol) in methanol (15 mL) was added 500 mg K_2_CO_3_. The suspension was stirred for 18 h at 24 °C. After which time, the solvent was evaporated, the residue was suspended in EtOAc (30 mL) and sequentially washed with water (15 mL) and brine (15 mL). The organic phase was dried over MgSO_4_ and evaporated to dryness. The residue was purified by silica gel column chromatography (0–2% MeOH/CH_2_Cl_2_, w 0.5% TEA). The pure product (**4**) was obtained as a solid foam (240 mg, 72%).^1^H NMR (400 MHz, CDCl_3_) δ 8.36 (s, 1 H, H2), 8.30 (s, 1 H, H8), 7.40–7.20 (m, 9 H, DMT), 6.82 (d, 4 H), 6.25 (dd, *J* = 2.0, 7.5 Hz, 1 H, H1′), 5.63 (s, 2 H, NH2), 4.51 (m, 1 H, H3′), 3.78 (s, 6 H, 2-OCH3), 3.75–3.65 (m, 2 H), 2.40–2.30 (m, 1 H, H2a′), 2.15 (m, 1 H, H2b′).

*N* ^*6*^-(ε-tertbutyldiphenylsilyloxy-hexanoyl)-9-[3′-*O*-[(4′,4″-dimethoxytriphenyl) methyl]-2′-deoxy-α-l-threofuranosyl]adenine (**5**). To a solution of **4** (240 mg, 0.46 mmol) and 6-O-TBDPS-hexanoic acid^50^ (**R1**) (347 mg, 0.94 mmol) in dry 1,2-dichloro-ethane was added dry DIPEA (0.5 mL, 2.8 mmol) and PyBOP (600 mg, 1.15 mmol). After stirring for 18 h at 70 °C, the solvents were evaporated and the crude material was purified by silica gel chromatography (20–80% EtOAc-Hexane containing 0.5 % TEA). The pure product (**5**) was obtained as a solid foam (175 mg, 43%).^1^H NMR (400 MHz, CDCl_3_) δ 8.67 (s, 1 H, H2), 8.44 (s, 1 H, H8), 8.39 (brs, 1 H, NH), 7.69–7.62 (m, 4 H, Ar), 7.4–7.18 (m, 15 H, Ar), 6.81 (d, 4 H, DMT), 6.30 (dd, *J* = 2.0, 7.5 Hz, 1 H, H1′), 4.52 (m, 1 H, H3′), 3.78 (s, 6 H, 2-OCH_3_), 3.77–3.70 (m, 2 H, H4a′, H4b′), 3.68 (t, 2 H, CH_2_), 2.87 (t, 2 H, CH_2_), 2.40–2.35 (m, 1 H, 2 Ha′), 2.13 (m, 1 H, H2b′), 1.82–1.44 (m, 6 H, 3xCH_2_), 1.04 (s, 9 H, tert-Bu).

*N* ^*6*^-(ε-hydroxy-hexanoyl)-9-[3′-*O*-[(4′,4″-dimethoxytriphenyl)methyl]-2′-deoxy-α-l-threofuranosyl]adenine **(6)** To a solution of **5** (175 mg, 0.20 mmol) in THF (4 mL) was added 1 M TBAF in THF (1 mL)^29^. After stirring for 30 min at 24 °C, the solvents were evaporated and the crude material was purified by silica gel chromatography. The pure product (**6**) was obtained as a solid foam (90 mg, 70%).^1^H NMR (400 MHz, CDCl_3_, Supplementary Fig. 8a) δ 8.85 (s, 1 H, NH), 8.70 (s, 1 H, H2), 8.46 (s, 1 H, H8), 7.40–7.20 (9 H), 6.82 (d, 4 H), 6.31 (dd, *J* = 1.5, 7.0 Hz, 1 H, H1′), 4.52 (m, 1 H, H3′), 3.79 (s, 7 H, 2-OCH_3_, H4a′), 3.71 (m, 3 H, H4b′, ε-CH2), 2.90 (t, 2 H, α-CH_2_), 2.33 (m, 1 H, H2a′), 2.18 (m, 1 H, H2b′), 1.90–1.80 (m, 2 H, β-CH_2_, OH), 1.70–1.60 (m, 2 H, δ-CH_2_), 1.60-1.50 (m, 2 H, γ-CH_2_);^13^C NMR (125 MHz, CDCl_3_, Supplementary Fig. 8b) δ 158.9, 152.6 (C2), 151.2, 149.2, 144.84, 141.7 (C8), 136.1, 130.0 (Ar), 128.3 (Ar), 128.0 (Ar), 127.3 (Ar), 122.2, 113.7 (DMT), 87.9, 84.8 (C1′), 75.2 (C4′), 72.9 (C3′), 62.8 (Cε), 55.4(OCH3), 39.6 (C2′), 37.8 (Cα), 32.3 (Cδ), 25.5 (Cγ), 24.8 (Cβ); HRMS (ESI-TOF): [M + Na]^+^ calcd for C_36_H_39_N_5_O_6_Na, 660.2798; found 660.2782.

Synthesis of 2′-deoxy-threofuranosyl adenosine-modified CPG **(7)**. To a solution of **6** (30 mg, 46 µmol) in dry DCM (1 mL) was added 0.5 M DIPEA/ DCM (200 mL) and 0.5 M 2-Cyanoethyl N,N-diisopropylchlorophosphoramidite in DCM (100 µL). After stirring for 1 h at 24 °C, the reaction was added to dried detritylated dT-CPG (**R2**) (150 mg) and 0.25 M ETT in MeCN (0.5 mL) and stirred for 15 min at 24 °C. The CPG was filtered, washed with DCM, and MeCN. The CPG was then placed into three DNA synthesis columns and oxidized and capped using standard DNA synthesis protocol at 1 µmol scale.

### Oligonucleotide synthesis

TNA modified oligonucleotides were synthesized on an Applied Biosystems 3400 DNA synthesizer using standard β-cyanoethyl phosphoramidite chemistry (Supplementary Table 1). The P1 primer was synthesized on a Universal Support II CPG column (1 μM scale, Glen Research) using chemically synthesized tA phosphoramidite^29^. The P2 primer was synthesized using the chemically synthesized 2′-deoxy-threofuranosyl adenosine-modified CPG (Fig. 2). TNA oligonucleotides were obtained by solid-phase synthesis on a 1-μmol scale using standard DNA coupling conditions^10^. Cleavage from the solid support and deprotection of the oligonucleotides was achieved in NH_4_OH (33%) for 18 h at 55 °C. Oligonucleotides were purified by preparative denaturing polyacrylamide gel electrophoresis, isolated, ethanol precipitated, and desalted on a sephadex G-25 ion exchange resin. Pure salt-free oligonucleotides were validated by MALDI-TOF mass spectroscopy, UV quantified, and stored in H_2_O at −20 °C.

### Kod-RI expression and purification

The *kod-RI* gene was PCR amplified from a previously constructed vector, pGDR11-Kod-RI^16^ which additionally harbors two mutations (D141A and E143A) to inactivate exonuclease activity, using Kod-RI_for and Kod-RI_rev primers (IDT) containing *NdeI* and *NotI* restriction enzyme sites, respectively (Supplementary Table 1). Purified PCR product and pET21 (Novagen) were digested with *NdeI* and *NotI* restriction enzymes (NEB) and ligated and the resulting pET21-*kod-RI* construct was sequence verified (Retrogen). Acella® cells (Edge BioSystems) harboring pET21-*kod-RI* were grown aerobically at 37 °C in LB medium containing 100 μg mL^−1^ ampicillin. At an OD_600_ of 0.8, expression of a tagless Kod-RI was induced with 0.8 mM isopropyl β-D-thiogalactoside at 18 °C for 20 hr. Cells were harvested by centrifugation for 20 min at 3315 × *g* at 4 °C and lysed in 40 mL lysis buffer (10 mM Tris.Cl pH 7.5, 100 mM NaCl, 0.1 mM EDTA, 1 mM DTT, 10% glycerol, 5 mg egg hen lysozyme) by sonication. The cell lysate was centrifuged at 23,708 × *g* for 30 min and the clarified supernatant was heat treated for 20 min at 70 °C and centrifuged again at 23,708 × *g* for 30 min. The supernatant was loaded onto 5 mL HiTrap Q HP and heparin HP columns (GE) assembled in series with the efflux of the Q column loaded in front of the heparin column. After washing with lysis buffer, the Q column was removed and Kod-RI was eluted from the heparin column with a high salt buffer (10 mM Tris.Cl pH 7.5, 1 M NaCl, 0.1 mM EDTA, 1 mM DTT, 10% glycerol) using a linear gradient. Eluted fractions containing Kod-RI were visualized by SDS-PAGE, pooled, and concentrated using a 30 kDa cutoff Amicon centrifugal filter (Millipore). Further purification was achieved by size exclusion chromatography (Superdex 200 HiLoad 16/600, GE) pre-equilibrated with Kod-RI buffer (50 mM Tris.Cl pH 8.5, 200 mM NaCl, 0.1 mM EDTA, 1 mM DTT). Purified Kod-RI was concentrated to 10 mg mL^−1^ for crystallization trials.

### TNA synthesis

Primer-extension reactions were performed in a final volume of 10 µl using the PBS8 primer (5′-IR800-label-GTCCCCTTGGGGATACCACC-3′) and the L11 library (5′-GGATCGTCAGTGCATTGAGA-N_40_-GGTGGTATCCCCAAGGGGAC-3′, where N is the random region). Each reaction contained 10 pmol primer/template complex, 1× ThermoPol buffer [20 mM Tris-HCl, 10 mM (NH4)_2_SO_4_, 10 mM KCl, 2 mM MgSO_4_, 0.1% Triton X-100, pH 8.8], 1 µM KOD-RI, 100 µM of each tNTP, and 1 mM MnCl_2_. Reactions were incubated for 30 min at 55 °C, quenched with stop buffer (8 M urea, 45 mM EDTA) and analyzed by 20% denaturing urea PAGE. The uncropped gel is provided as Supplementary Fig. 9.

### Michaelis–Menten kinetic analysis

Kinetic measurements were performed in 96-well format. Each measurement (10 μL) contained a final concentration of 1 μM of the self-priming hairpin template (5′-TCTCTATAGTGAGTCGTATAGGT GGTATCCGAAAGGATACCACC-3′), 1× Thermopol buffer, 4× Eva Green fluorescent dye, 1 mM MnCl_2_, 1 μM Kod-RI, and a titrated concentration of tNTPs [2.5–100 μM]. Reactions were initiated by denaturing for 2 min at 95 °C, and extended for 2 h at 55 °C with fluorescence intensity measurements collected at 6 s intervals. The first 18 s of data were excluded to eliminate possible artifacts caused by temperature equilibration. The data were fit using a nonlinear regression in R using the equation *Y* = (*V*_min_ + *V*_max_×*X*×(*X* + *K*_m_)^−1^ where *V*_min_ is the minimum velocity and *V*_max_ is the maximum velocity. The reported values derive from the average of three independent replicates with error bars defining the standard deviation.

### *K*_D_ analysis

A twofold titration series starting from 2 μM Kod-RI was generated in 1× Thermopol buffer in a final volume of 5 μL. The polymerase solution was combined with 5 μL of annealed primer–template complex [TNA strand: 5′-gacactcgtatgcagtagcc-3′ 5′-labeled DNA strand: 5′-Cy5 ACATTGGCATCAAGTCATAA GGCTACTGCATACGAGTGTC-3′] at a final concentration of 2 nM in 1× Thermopol buffer. The reaction mixtures were then incubated for 60 min at 23 °C. The samples were then loaded into separate capillaries and loaded onto the Monolith NT.115 Pico (Nanotemper). The capillaries were scanned using 20% laser and MST power and the data was plotted. The dissociation constant (*K*_D_) was determined using a standard fit using custom software from Nanotemper. Control experiments with wild-type Kod and engineered Kod-RI binding to DNA/DNA and DNA/TNA duplexes are provided in Supplementary Fig. 10.

### Crystallization procedures

All reagents purchased from commercial suppliers were of analytical grade. Stock solutions of sodium sulfate decahydrate (Fluka), polyethylene glycol 3000 and 3350 (Sigma Aldrich), and 2-(N-morpholino)ethanesulfonic acid (Calbiochem) were filtered before use. The additives, 1,6-Hexanediol and Silver Bullets Bio #56 (Hampton Research), were used without further manipulation. All crystallization samples were prepared according to the desired mechanistic step (see below) and 0.2 μL appropriate additive (10% v/v of 2 μL crystallization drop) was added before centrifugation. 24-well plate hanging drop trays were used to optimize crystals over a range of pH and PEG concentration, with every drop containing 1 μL of sample mixed with 1 μL of mother liquor over 500 μL mother liquor in every well. Trays were stored in the dark at room temperature. Crystals typically grew between 1–4 weeks.

### Duplex preparation

The DNA template (Supplementary Table 1) was purchased from IDT as an HPLC purified sample bearing a 5′ Cy5 label. The template strand (T) was used without further purification as a substrate for crystallization trials^31, 51^. Duplexes of P1/T and P2/T were prepared by combining equal amounts of the primer and template strands in Kod-RI buffer (see protein expression) supplemented with 20 mM MgCl_2_, and annealing the strands by heating at 95 °C for 5 min and slowing cooling to 10 °C over 10 min.

### Crystallization and structure determination

All crystals were grown in hanging drops and transferred into mother liquor containing 20% glycerol (or the corresponding polyethylene glycol concentrations in the respective crystallization conditions) immediately before harvesting. The specific crystallization conditions for each polymerase state are described below. Five diffraction data sets were collected at synchrotron sources (Advanced Light Source and Stanford Synchrotron Radiation Lightsource) from single crystals. Unless specified, images were indexed, integrated, and merged using iMOSFLM^52^. Data collection statistics are summarized in Table 1. Initial models were determined by molecular replacement (MR) using Phaser^53^ and all final models were determined using iterative rounds of manual building through Coot^54^ and refinement with phenix^55^. The final stages of refinement employed TLS parameters; unless specified, Kod-RI was partitioned into 4 TLS groups (i.e., 1–156, 157–304, 305–532, 533–756) while the template and primer strands contributed an additional TLS group each. The stereochemistry and geometry of all structures were validated with Molprobity^56^, with the final refinement parameters summarized in Table 1. Final coordinates and structure factors have been deposited in the Protein Data Bank. All molecular graphics were prepared with PyMOL^57^.

*Apo Kod-RI*: Apo Kod-RI (1 mg mL^−1^) crystallized in 0.1 M 2-(N-morpholino)ethanesulfonic acid pH 6.0, and 20% polyethylene glycol 3000. MR was performed using Kod^exo-^ (exonuclease deficient Kod: D141A and E143A, PDB ID: 1WNS) as the search model^33^. The final apo Kod-RI model contains unbuilt regions in the thumb domain due to poor electron density (i.e., residues 610–617, 667–677, 688–698, 704–712, 716–718, 722–725, and 747–748). PDB ID: 5VU5.

*Binary complex:* The binary complex was prepared by incubating Kod-RI (5 mg mL^−1^) with 1.5 molar equivalents of the P1/T duplex at 37 °C for 30 min. The binary complex co-crystallized in 0.2 M sodium sulfate decahydrate, 0.1 M 2-(N-morpholino)ethanesulfonic acid pH 6.0, and 16 % polyethylene glycol 3350, supplemented with Silver Bullets Bio (Hampton Research) additive #56 (0.2 % w/v D-Sorbitol, 0.2 % w/v Glycerol, 0.2 % w/v Glycine, 0.2% w/v myo-Inositol, 0.2 % w/v Sarcosine, 0.02 M HEPES sodium pH 6.8). MR was performed using the Kod^exo-^ binary complex structure (PDB ID: 4K8Z) as the search model in which the P/T duplex sequence is identical except for a single TNA A residue (tA12, Supplementary Table 1) at 3′ end of the primer strand^31^. After MR, a tA model replaced the corresponding deleted A12 and its phosphate group was linked to the preceding O3′ of G11 before iterative refinement and model building began. PDB ID: 5VU6.

*Ternary complex (open)*: An initial binary complex was prepared by incubating Kod-RI (5 mg mL^−1^) with 1.5 M molar equivalents of the P2/T duplex at 37 °C for 30 min. 5 M excess of tATP monomer was added to the binary complex and the solution was incubated at 37 °C for 30 min. The ternary complex co-crystallized in 0.2 M sodium sulfate decahydrate, 0.1 M 2-(N-morpholino)ethanesulfonic acid pH 5.5, 3% w/v 1,6-Hexanediol, and 22 % polyethylene glycol 3350. Ternary complex crystals were transferred to mother liquor containing 20% glycerol additionally supplemented with 2 mM tATP for 30 min. MR was performed using an early binary complex (with P1/T duplex) structure as the search model. tATP was initially included as the incoming substrate; however, due to poor electron density, tATP was replaced by tA. The final open ternary complex structure includes, besides tA, one sulfate ion and one magnesium ion. PDB ID: 5VU7.

*Ternary complex (closed)*: The closed ternary complex crystals were prepared using a similar protocol to the open ternary complex crystals with the P2/T duplex. 2 mM tATP and 20 mM MgCl_2_ were directly added to the crystals grown in 0.2 M sodium sulfate decahydrate, 0.1 M 2-(N-morpholino)ethanesulfonic acid pH 4.0, 3 % w/v 1,6-Hexanediol, and 19 % polyethylene glycol 3350 for 45 min. The images were indexed, integrated, and merged using HKL2000^58^ and MR was performed using an early open ternary complex structure, with tATP as its substrate, as the search model. During initial model building, the finger domain was deleted and rebuilt based on finger domain of the structurally aligned yeast Pol δ (PDB ID: 3IAY)q^24^. The final closed ternary complex structure spans residues 1–759 and contains a total of 23 residues mutated to alanines (i.e., E150, E154, E200, K221, Q285, R346, E385, R394, R476, I528, K531, K638, R668, L704, K705, I710, R713, H725, K726, Y727, D728, Q736, and R751) as well as two missing residues (i.e., E658 and R689) due to poor electron densities and three magnesium ions. PDB ID: 5VU8.

*Translocated product*: The binary complex with the P1/T duplex was prepared using a similar protocol and 5 M excess tATP was added and incubated at 37 °C for 30 min before crystallization. Co-crystals of the translocated complex were grown in 0.2 M sodium sulfate decahydrate, 0.1 M 2-(N-morpholino)ethanesulfonic acid pH 5.8, and 12 % polyethylene glycol 3350, supplemented with Silver Bullets Bio (Hampton Research) additive #56. MR was performed using an early binary complex (with P1/T duplex) structure as the search model. During initial model building, the P1/T duplex was translocated and a TNA A model, tA13, was added and its phosphate group linked to 2′-O atom of tA12. The final translocated product structure spans residues 1–757 and contains 363 water molecules. PDB ID: 5VU9.

### Data availability

Coordinates and structure factors for all five Kod-RI crystal structures have been deposited in the PDB with the accession codes: 5VU5, 5VU6, 5VU7, 5VU8, and 5VU9. Other data are available from the corresponding author upon reasonable request.

## Electronic supplementary material

Supplementary Information
