# Supplementary material for: Structural basis for TNA synthesis by an engineered TNA polymerase
Source: Nat Commun. 2017 Nov 27;8:1810. doi: 10.1038/s41467-017-02014-0 (PMC5703726; doi:10.1038/s41467-017-02014-0)
Supplement: Supplementary file 1 — Supplementary Information [file 41467_2017_2014_MOESM1_ESM.pdf]

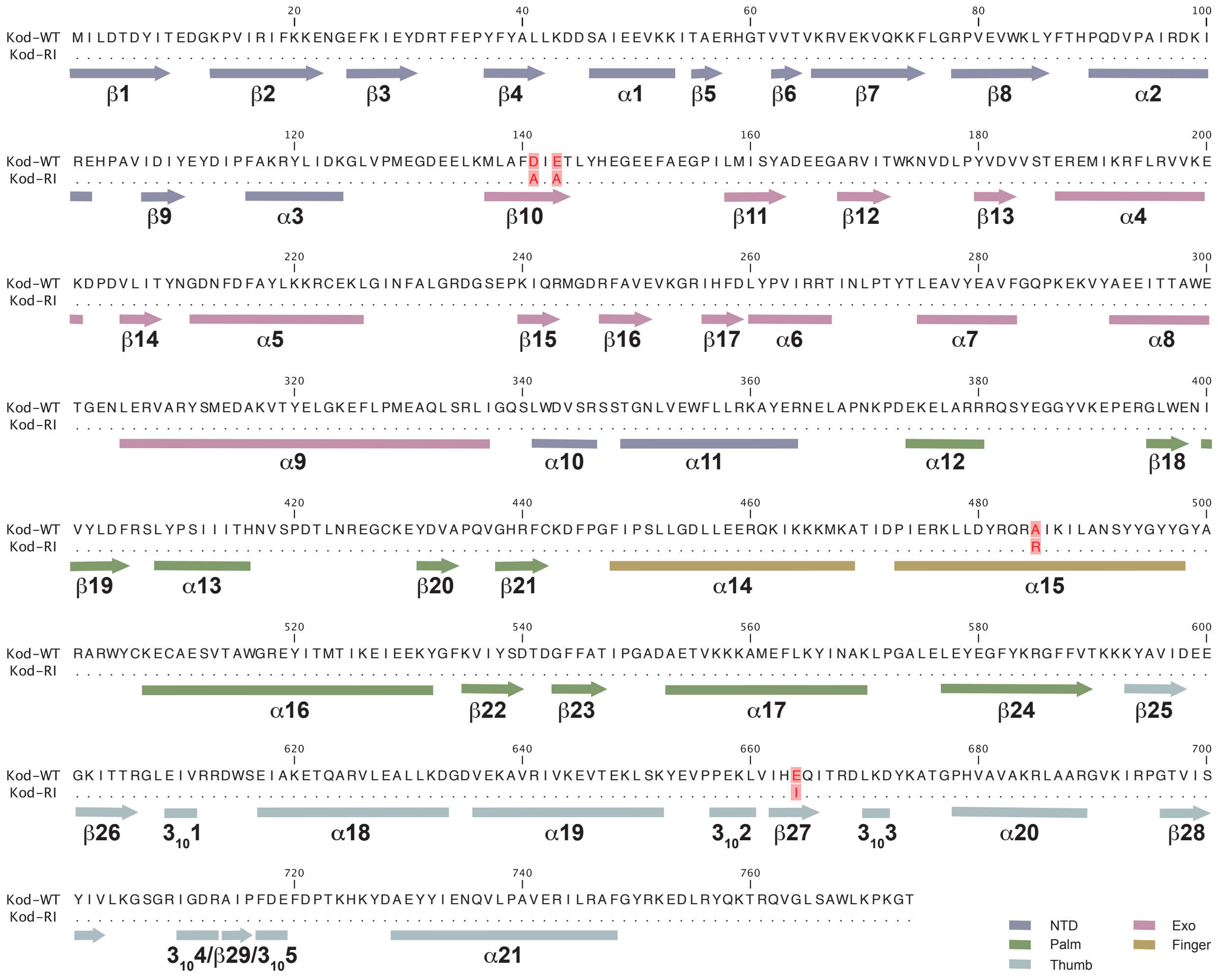

Supplementary Figure 1 Kod-WT and Kod-RI sequence alignment. Sequence differences between Kod-WT and Kod-RI are highlighted in red. Secondary structural elements are colored by domain as illustrated in **Figure 1** (main text).

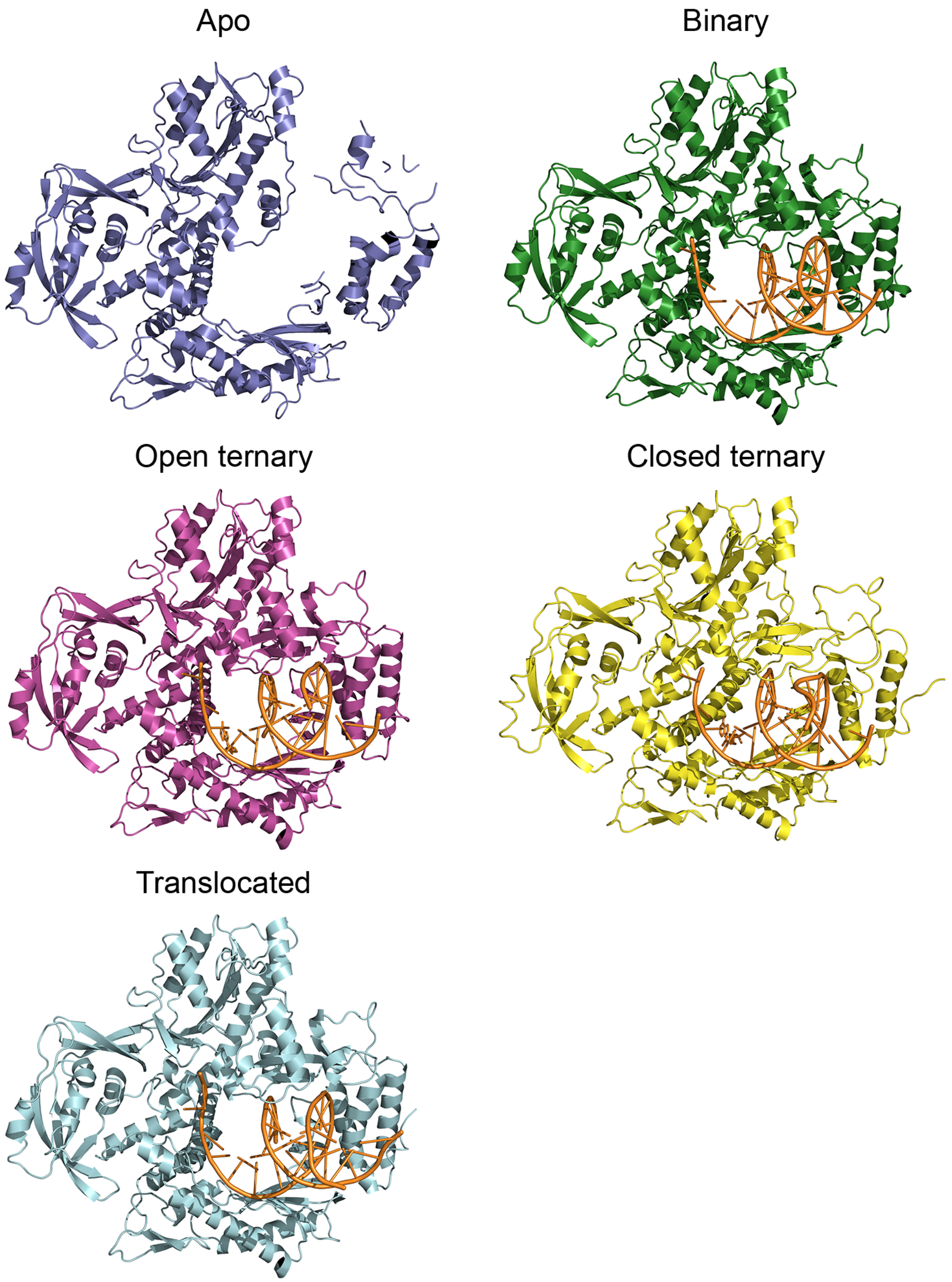

Supplementary Figure 2 X-ray crystal structures of Kod-RI TNA polymerase. Cartoon representation of apo, binary, open and closed ternary, and translocated structures. Primer-template duplex shown in orange.

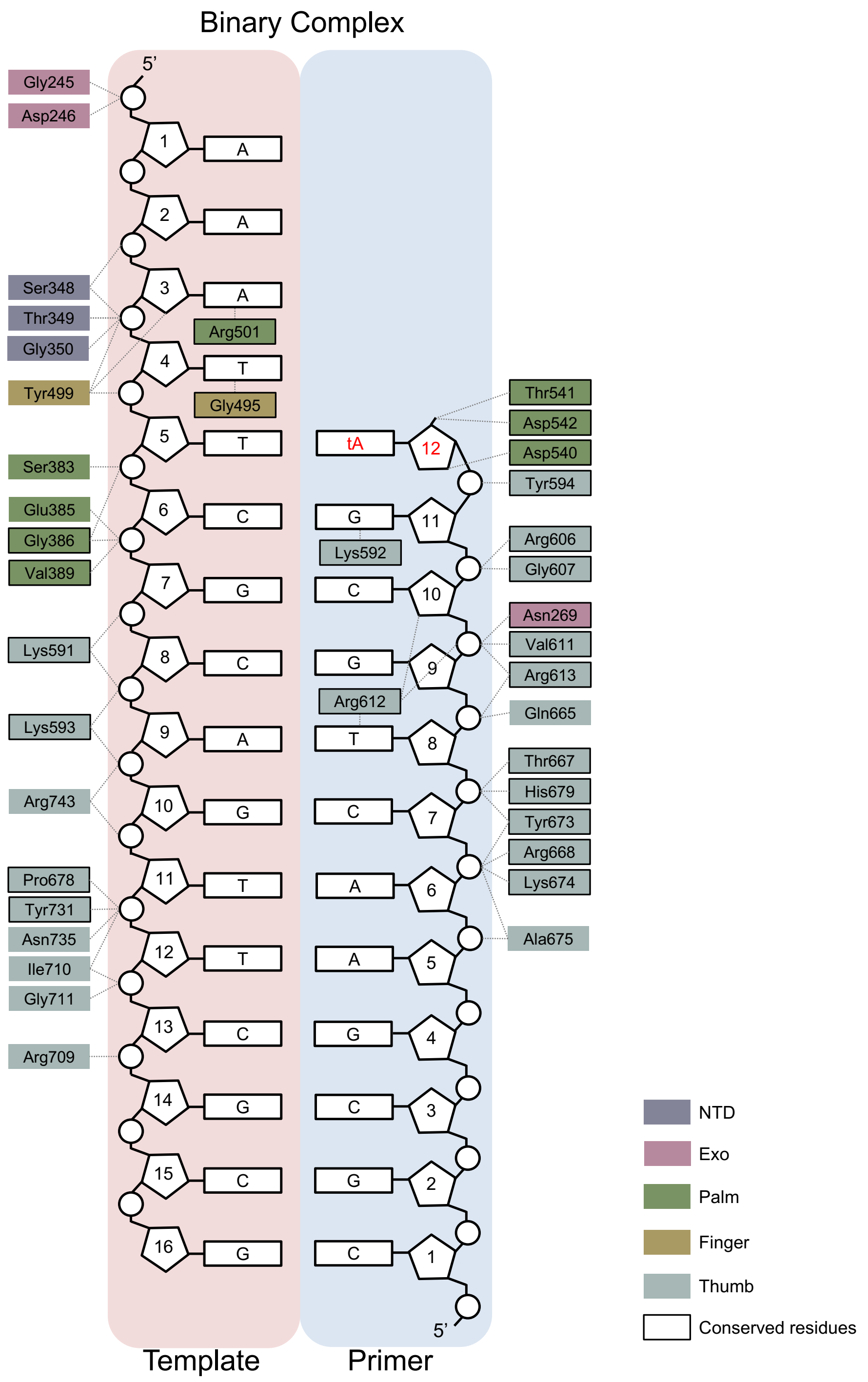

Supplementary Figure 3a Two-dimensional interaction map for the binary complex. Dashed lines show interactions between residues and their respective nucleotide moiety (i.e., phosphodiester backbone, sugar, or nucleobase). Residues are colored by domain as illustrated in **Fig. 1**. Conserved residues are boxed.

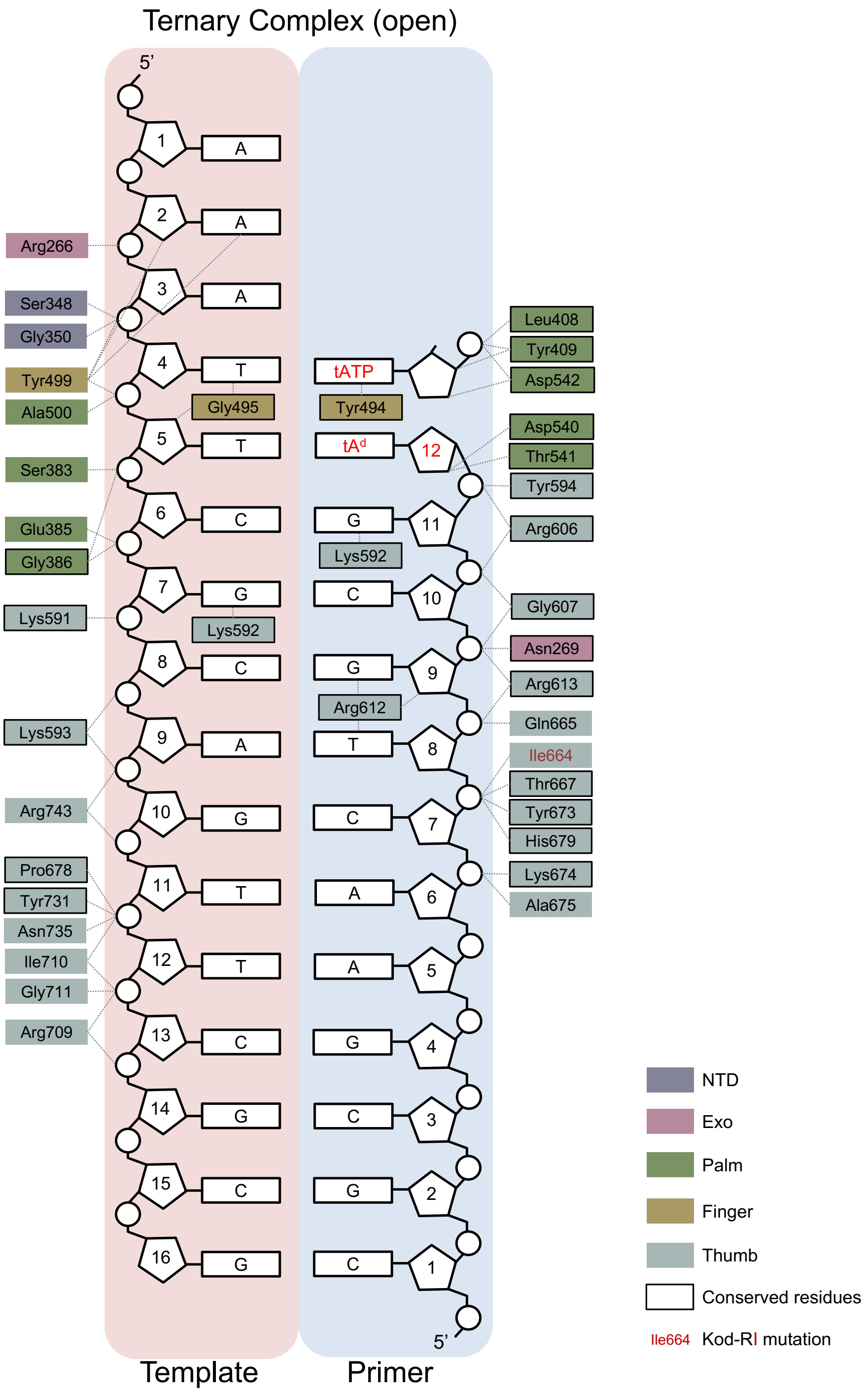

Supplementary Figure 3b Two-dimensional interaction map for the open ternary complex.

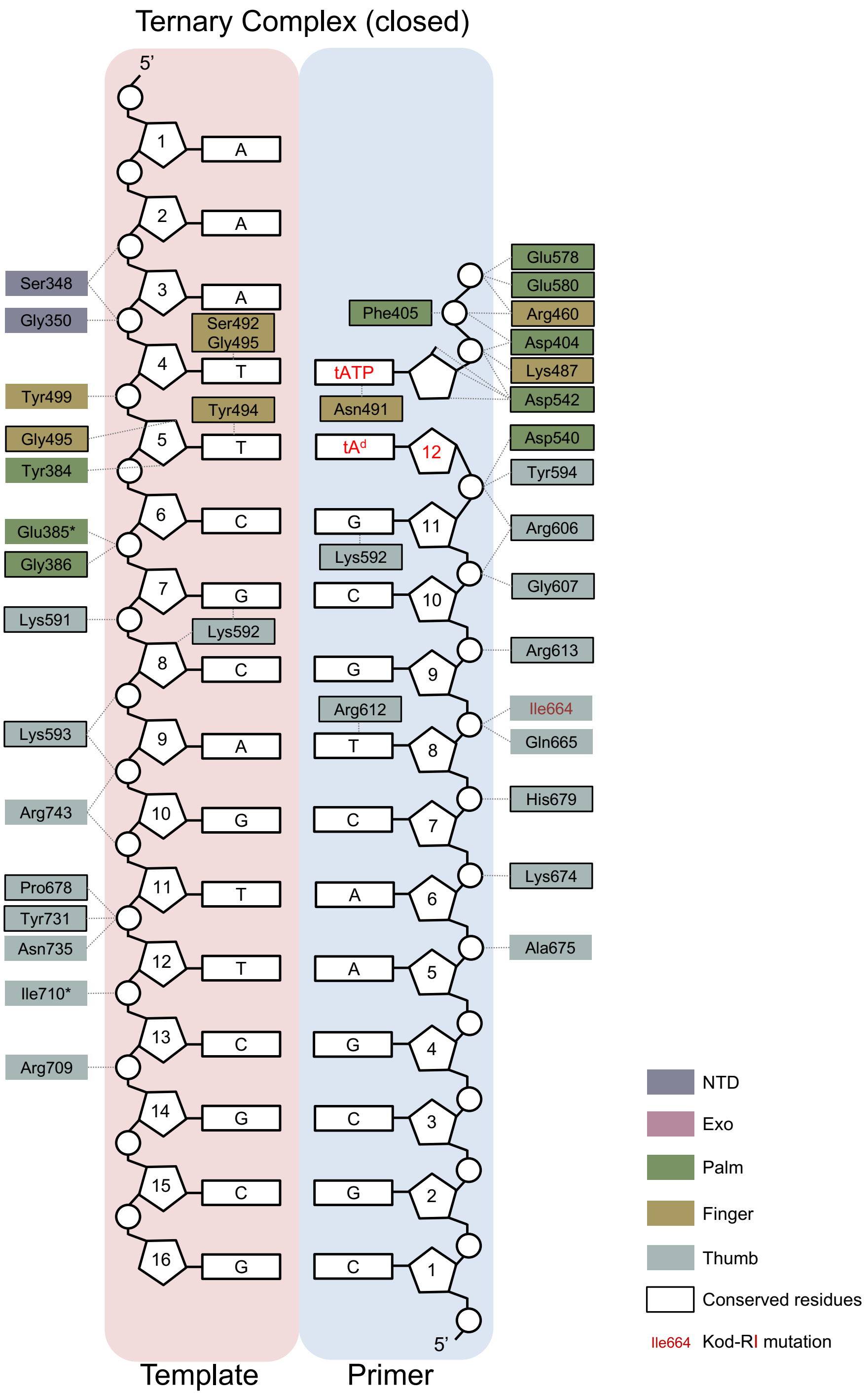

Supplementary Figure 3c Two-dimensional interaction map for the closed ternary complex. Asterisks denote residues that were mutated to alanine during structure refinement.

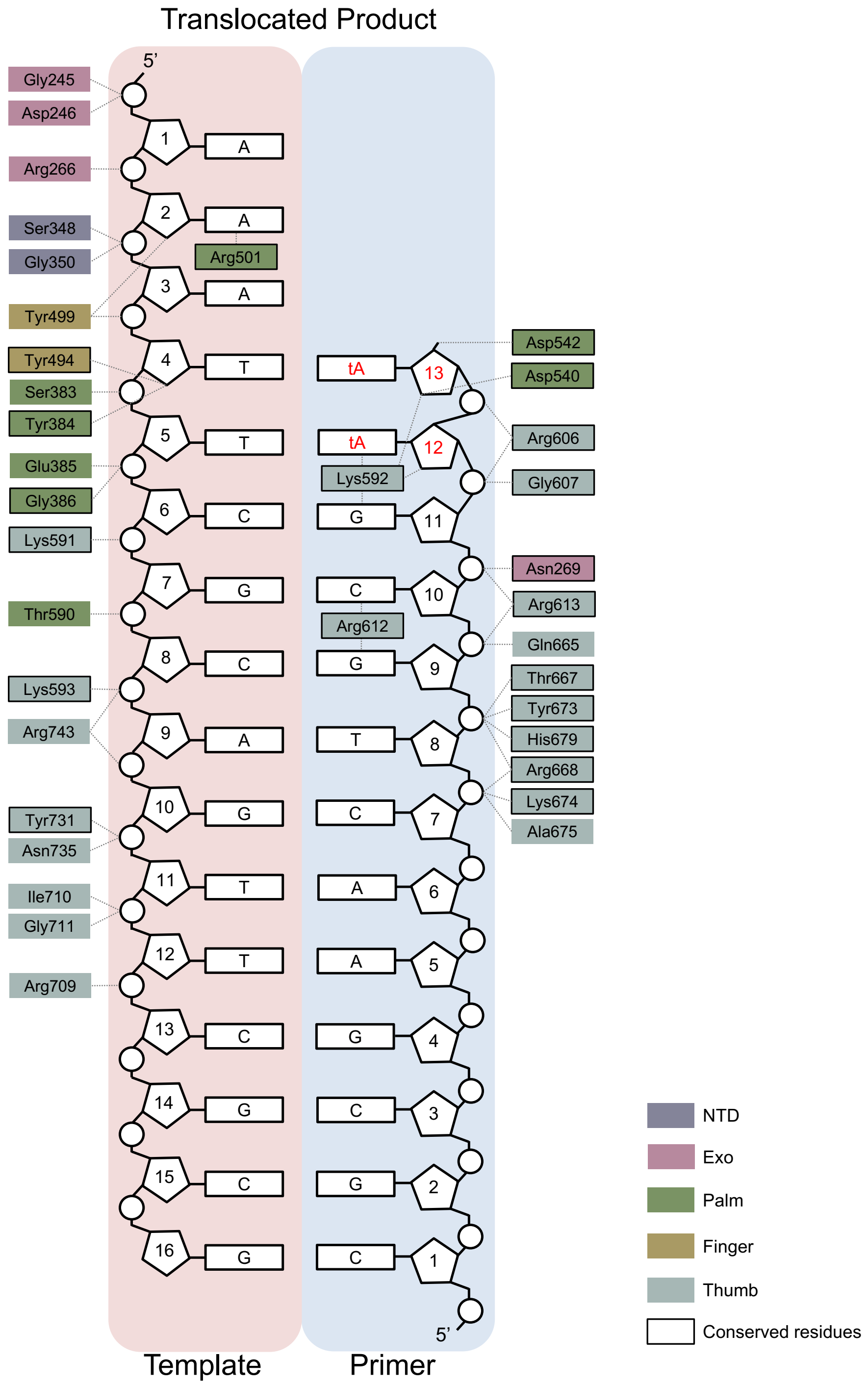

Supplementary Figure 3d Two-dimensional interaction map for the translocated product.

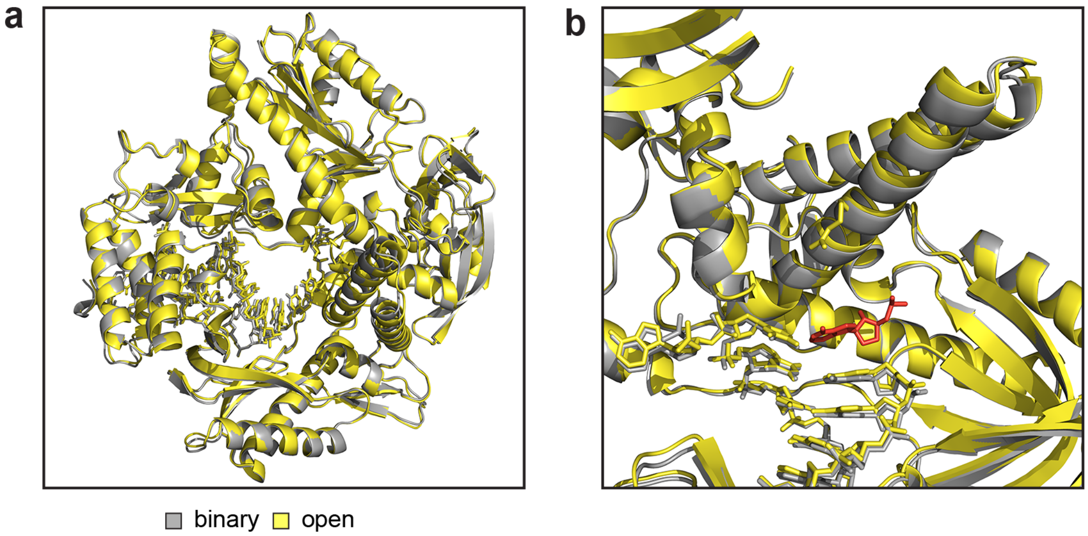

Supplementary Figure 4 Binary and open ternary structures adopt an open conformation. Structural overlay of the (**a**) global architecture and (**b**) active site of the binary (grey) and open ternary (yellow) complexes reveal minimal structural conformational change.

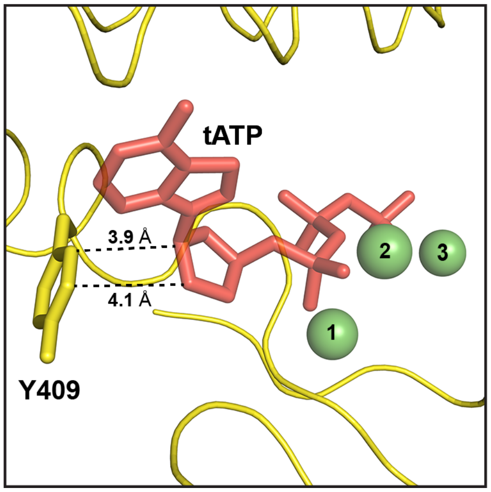

Supplementary Figure 5 Tyr409 does not interact with tATP. The distances between Tyr409 CE2 and tATP O4’ (4.1 Å) and Tyr409 CD2 and tATP C1’ (3.9 Å) are provided.

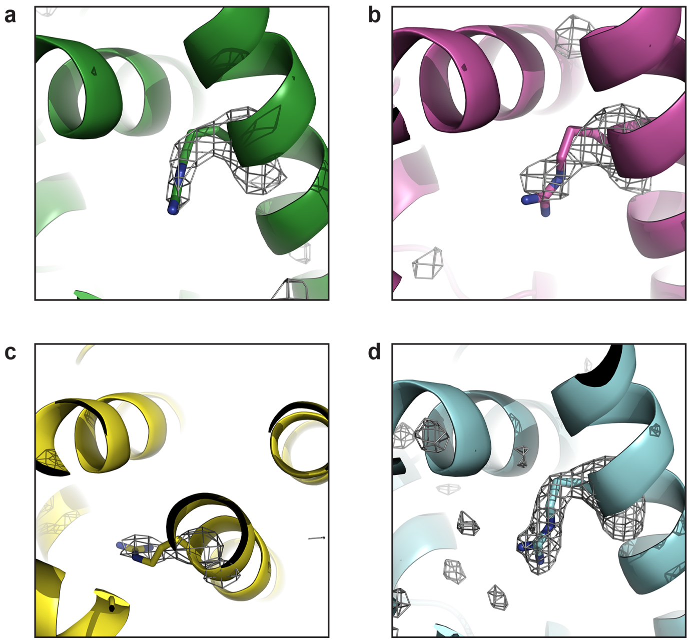

Supplementary Figure 6 Arg485 adopts distinct orientations in open and closed conformations. Simulated annealing Fo-Fc omit maps contoured at 3.0 σ for Arg485 from the (**a**) apo, (**b**) open and (**c**) closed ternary and the (**d**) translocated product structures.

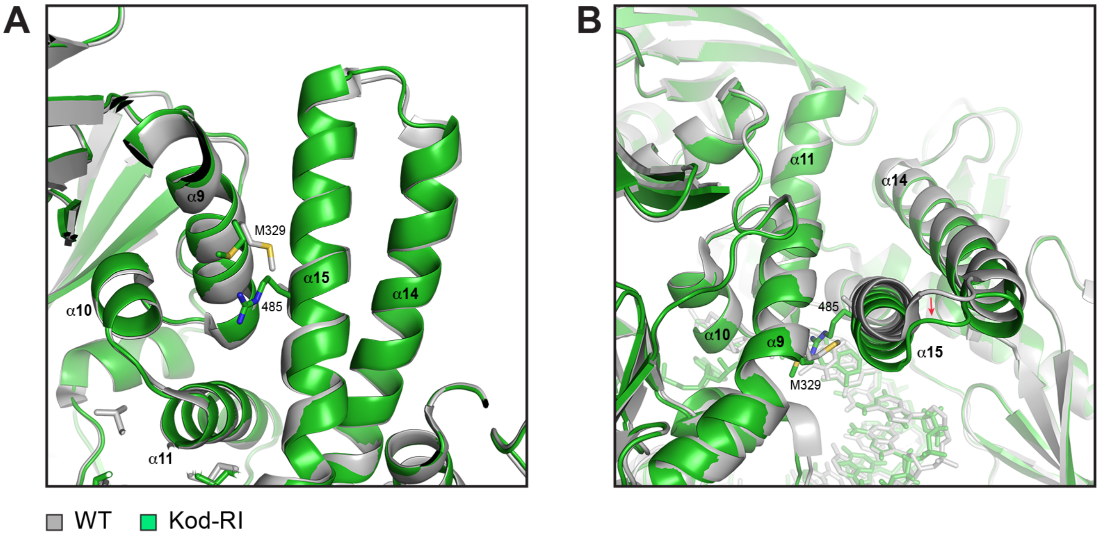

Supplementary Figure 7 Mutational consequence of Ala485Arg. Structural overlay of Kod-WT binary complex (grey) and Kod-RI translocated product complex (green) from the side (**a**) or top (**b**) view of the finger subdomain reveals repositioning of α15 and residue Met329.

a

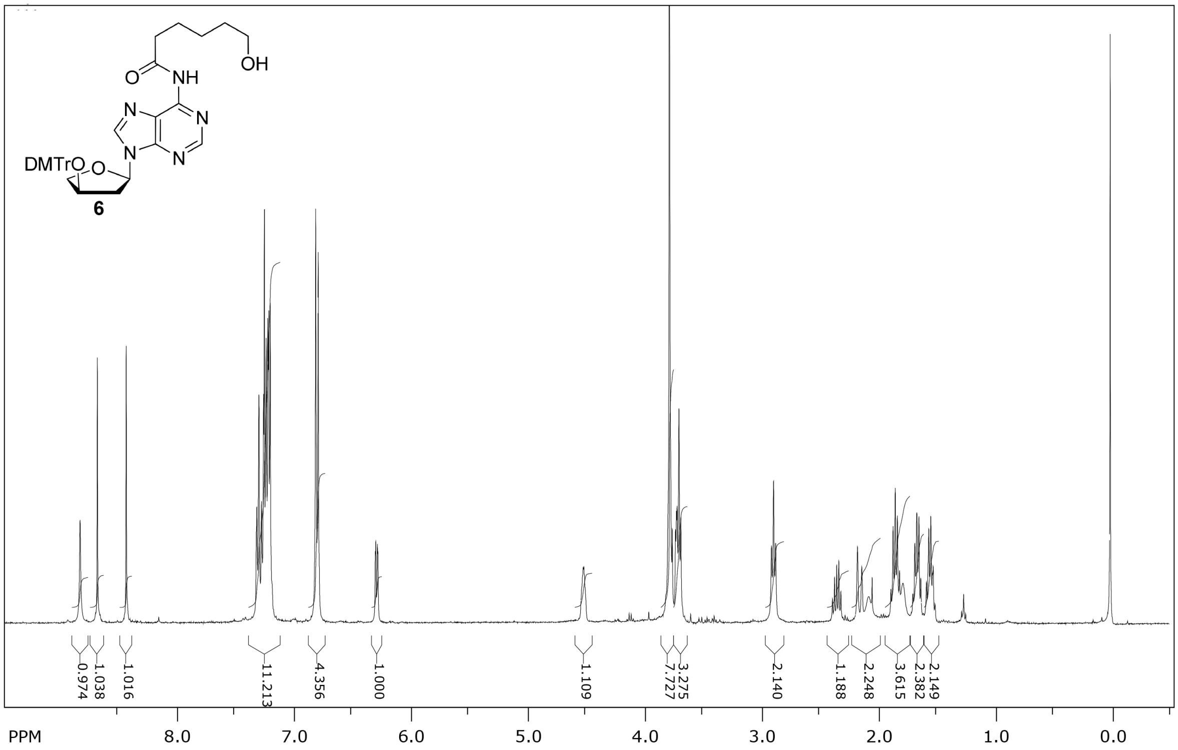

b

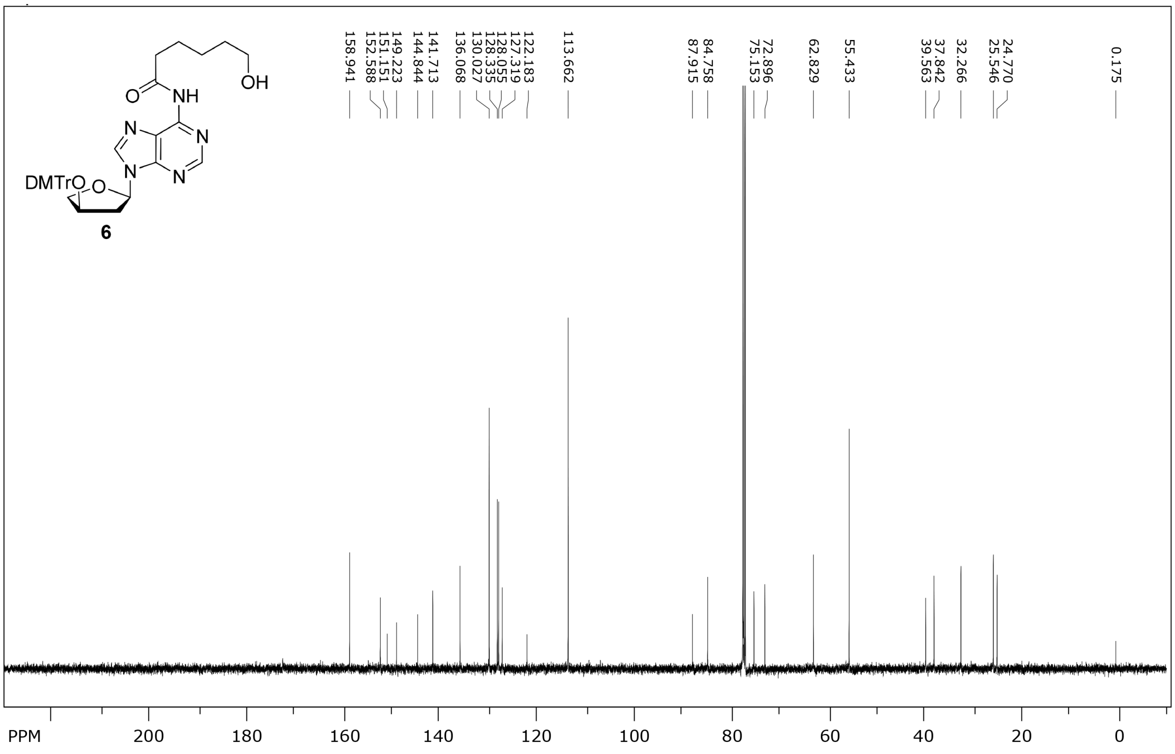

Supplementary Figure 8 Validation of compound 6 (**a**) ^{1}H-NMR and (**b**) ^{13}C-NMR spectra of compound 6, collected at 400 MHz in $CDCl_3$.

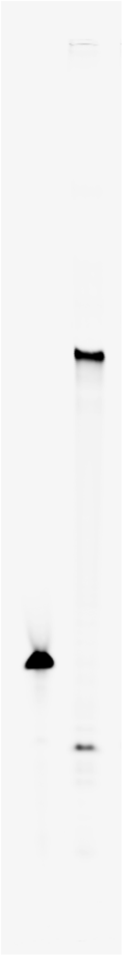

Supplementary Figure 9. Uncropped gel image from Figure 1b.

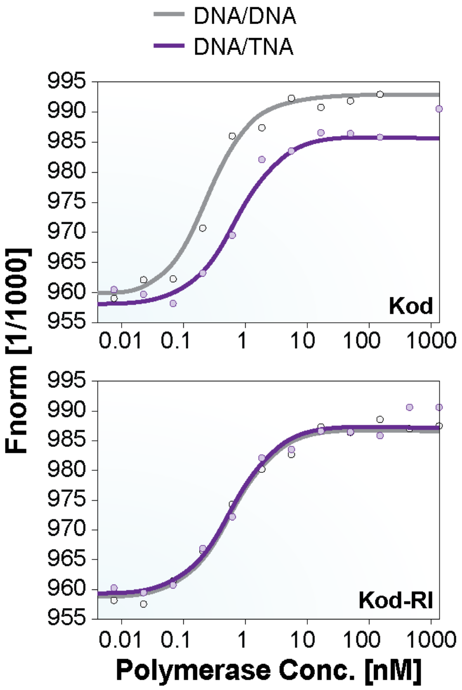

Supplementary Figure 10. Full dataset acquired for the polymerase duplex binding assay. K_d binding profiles observed for Kod wild-type and engineered Kod-RI bound to the natural DNA/DNA duplex and the chimeric DNA/TNA duplex.

Supplementary Table 1 Oligonucleotide sequences.

Oligonucleotide name	Oligonucleotide sequence (5' – 3')	Comments
**Kod-RI_for**	ATC*CATATG*ATCCTCGACACTGACTAC	*NdeI*, cloning
**Kod-RI_rev**	ACGCAT*GCGGCCGC*TCAAGTTCCCTTCGGCTTCAGCCA	*NotI*, cloning
**PBS8**	GTCCCCTTGGGGATACCACC	IR800 modified at 5', TNA synthesis
**L11**	GGATCGTCAGTGCATTGAGA-N_{40}-GGTGGTATCCCCAAGGGGAC	N refers to any nucleotide, TNA synthesis
**Hairpin template**	TCTCTATAGTGAGTCGTATAGGTGGTATCCGAAAGGATACCACC	Kinetic analysis
**TNA strand**	gacactcgtatgcagtagcc	Lower case for TNA, K_D
**DNA strand**	ACATTGGCATCAAGTCATAAGGCTACTGCATACGAGTGTC	Cy5 modified at 5', K_D
**Template (T)**	AAATTCGCAGTTCGC	Cy5 modified at 5'
**Primer 1 (P1)**	CGCGAACTGCG*tA*	TNA A (tA) at 3'
**Primer 2 (P2)**	CGCGAACTGCG*tA*^{d}	2'-deoxy TNA-A (tA^d) at 3'

Supplementary Table 2 Base pair statistics for Kod structures

	Binary	Ternary (open)	Ternary (closed)	Translocated Product	Kod^{exo-} (Binary)
**1. T4**	**-**	**tATP**	**tATP**	**tA13**	**-**
Shear (Å)		2.05	-0.23	-0.25	
Stretch (Å)		-0.13	-1.02	-0.37	
Stagger (Å)		1.03	-1.48	0.29	
Buckle (°)		-31.67	-17.76	-16.69	
Propeller (°)		-32.94	-29.04	-15.14	
Opening (°)		0.66	-24.77	1.70	
**2. T5**	**tA12**	**tA12**	**tA12**	**tA12**	**A12**
Shear (Å)	-1.11	-0.35	-1.34	-0.30	-0.29
Stretch (Å)	0.01	-0.03	-0.14	-0.20	-0.05
Stagger (Å)	0.31	0.39	-0.27	-0.32	-0.01
Buckle (°)	-22.22	-31.43	-33.62	-4.28	-22.81
Propeller (°)	-17.52	-19.58	-8.86	-4.75	-15.12
Opening (°)	9.83	5.53	-5.73	1.76	2.77
**3. C6**	**G11**	**G11**	**G11**	**G11**	**G11**
Shear (Å)	0.12	0.02	0.58	0.37	0.09
Stretch (Å)	-0.09	-0.15	-0.57	-0.25	-0.18
Stagger (Å)	-0.20	0.29	0.38	-0.22	-0.20
Buckle (°)	-12.05	-24.65	-15.38	-5.48	-6.19
Propeller (°)	2.61	6.82	8.69	-4.14	4.29
Opening (°)	0.71	-0.49	4.14	1.47	-0.71
**4. G7**	**C10**	**C10**	**C10**	**C10**	**C10**
Shear (Å)	0.07	-0.07	0.28	-0.05	-0.23
Stretch (Å)	-0.66	-0.13	-0.13	-0.03	-0.05
Stagger (Å)	0.36	-0.54	-0.27	-0.26	-0.22
Buckle (°)	-6.97	-13.81	-6.31	-19.19	-11.52
Propeller (°)	-12.97	-17.07	-4.96	-5.24	-19.09
Opening (°)	-0.62	3.74	7.65	1.92	6.62
**5. C8**	**G9**	**G9**	**G9**	**G9**	**G9**
Shear (Å)	0.20	0.20	-0.19	0.22	0.15
Stretch (Å)	-0.44	0.03	0.90	-0.19	-0.18
Stagger (Å)	0.16	-0.10	0.47	-0.11	-0.08
Buckle (°)	-13.58	-12.85	-10.89	2.61	-10.97
Propeller (°)	1.76	-1.18	-5.01	-3.50	-6.91
Opening (°)	3.54	1.76	24.51	-1.07	1.91

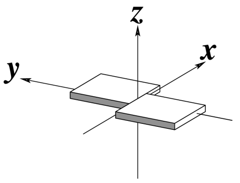

Shear

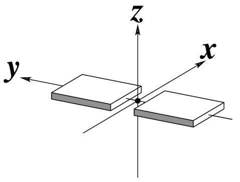

Stretch

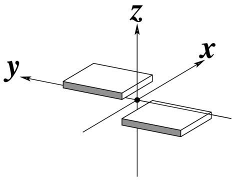

Stagger

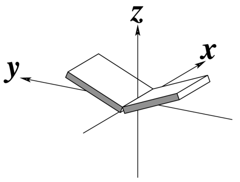

Buckle

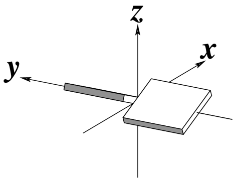

Propeller

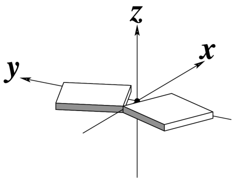

Opening

Supplementary Table 3 Base pair statistics for B-family polymerase ternary complexes

	Kod-RI open	Kod-RI closed	RB69	Phi29 (2PYL)	Phi29 (2PYJ)	Polα	Polδ	Polε*
**0**	**T:tATP**	**T:tATP**	**A:dTTP**	**A:dTTP**	**C:dGTP**	**C:dGTP**	**G:dCTP**	**T:dATP**
Shear (Å)	2.05	-0.23	0.02	0.00	0.14	0.53	0.13	-0.10
Stretch (Å)	-0.13	-1.02	-0.17	-0.07	-0.05	-0.17	-0.18	-0.16
Stagger (Å)	1.03	-1.48	-0.31	-0.08	0.08	-0.70	-0.05	-0.07
Buckle (°)	-31.67	-17.76	-11.64	-1.77	-0.43	13.32	2.09	1.52
Propeller (°)	-32.94	-29.04	-4.82	-2.28	-10.37	-14.62	-2.89	-9.71
Opening (°)	0.66	-24.77	3.16	-3.23	0.15	7.80	0.09	-0.51
**-1**	**T:tA**	**T:tA**	**G:C**	**T:A**	**G:C**	**C:G**	**G:C**	**G:C**
Shear (Å)	-0.35	-1.34	-0.70	0.08	0.33	-0.08	0.35	0.28
Stretch (Å)	-0.03	-0.14	-0.25	-0.12	-0.17	0.04	-0.16	-0.09
Stagger (Å)	0.39	-0.27	-0.40	-0.22	0.13	-0.14	-0.13	0.05
Buckle (°)	-31.43	-33.62	-9.90	-0.87	1.95	3.11	7.83	3.40
Propeller (°)	-19.58	-8.86	-5.10	-12.86	-13.09	-12.42	-9.59	-15.84
Opening (°)	5.53	-5.73	3.53	5.35	2.39	0.64	0.65	1.29
**-2**	**C:G**	**C:G**	**G:C**	**G:C**	**T:A**	**C:G**	**T:A**	**A:U^{I}**
Shear (Å)	0.02	0.58	-0.36	0.21	0.01	0.59	0.11	-0.12
Stretch (Å)	-0.15	-0.57	-0.03	-0.04	-0.03	-0.03	-0.11	-0.09
Stagger (Å)	0.29	0.38	-0.39	-0.16	-0.24	-0.36	-0.19	-0.08
Buckle (°)	-24.65	-15.38	2.17	-1.97	-5.34	20.74	-0.68	-2.99
Propeller (°)	6.82	8.69	-0.80	-3.79	-3.69	-9.97	1.31	-0.41
Opening (°)	-0.49	4.14	-1.12	-0.36	-0.30	-1.18	-1.02	-1.25
**-3**	**G:C**	**G:C**	**T:A**	**T:A**	**A:T**	**C:G**	**A:T**	**A:U^{I}**
Shear (Å)	-0.07	0.28	-0.07	-0.07	-0.10	0.87	-0.09	-0.19
Stretch (Å)	-0.13	-0.13	-0.11	-0.17	-0.14	-0.03	-0.07	-0.12
Stagger (Å)	-0.54	-0.27	-0.28	-0.20	-0.25	-0.05	-0.18	-0.07
Buckle (°)	-13.81	-6.31	0.83	-1.75	6.14	3.58	6.21	2.02
Propeller (°)	-17.07	-4.96	-6.47	-8.22	-7.17	-23.67	-3.17	-20.42
Opening (°)	3.74	7.65	1.07	4.47	3.91	8.85	0.10	3.65
**-4**	**C:G**	**C:G**	**A:T**	**A:T**	**A:T**	**T:A**	**G:C**	**C^{I}/G**
Shear (Å)	0.20	-0.19	-0.01	0.18	-0.01	-0.44	0.24	-0.29
Stretch (Å)	0.03	0.90	0.01	-0.16	-0.12	0.03	-0.16	-0.07
Stagger (Å)	-0.10	0.47	-0.14	-0.60	-0.07	0.26	-0.12	-0.10
Buckle (°)	-12.85	-10.89	-4.36	5.26	-7.35	-2.83	1.42	5.75
Propeller (°)	-1.18	-5.01	-2.11	-6.62	-14.35	12.47	-1.66	-13.69
Opening (°)	1.76	24.51	-2.23	1.08	2.16	-0.19	-2.61	2.99

*Polε contains several modifications within duplex: superscript I denotes Iodo-derivatized nucleotides

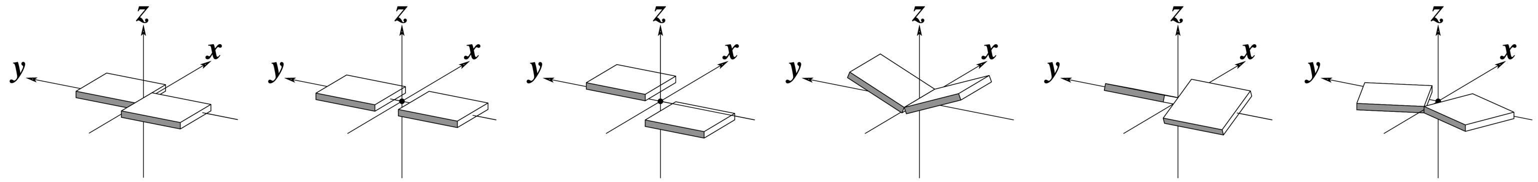

Shear Stretch Stagger Buckle Propeller Opening
